# Who leads matters: A governance-informed typology for implementing and scaling nature-based solutions

**DOI:** 10.1007/s13280-026-02421-y

**Published:** 2026-07-10

**Authors:** Kirk B. Enu, Christian Albert, Beate Jessel

**Affiliations:** 1 School of Architecture, Civil and Environmental Engineering, Laboratory of Landscape Development, Swiss Federal Technology Institute of Lausanne (EPFL), Station 16, 1015 Lausanne, Switzerland; 2 Institute of Environmental Planning, Leibniz University Hannover, Herrenhaeuser Str. 2, 30419 Hannover, Germany

**Keywords:** Actor agency, Archetypes, Governance, Implementation leadership, Institutional design, Policy instrument

## Abstract

Existing nature-based solutions (NbS) typologies prioritize technical form, ecosystem function or spatial scale and treat governance as secondary, limiting their ability to explain variation in feasibility, coordination, persistence and scaling. We propose a governance-informed typology anchored in implementation leadership—defined as the actor or coalition in which constitutive levers (decision authority, resource control and stewardship responsibility) converge at institutional stabilization. Operationalized through four analytical levers and applied to 35 urban NbS cases, the framework identifies five recurring governance archetypes: state-led, market-led, co-led, community-led and citizen-led. Leadership type is best inferred from the alignment of constitutive levers rather than from initiation. The typology complements existing classifications by demonstrating that seemingly similar interventions diverge substantially in governance structure and enabling conditions, with direct implications for policy instrument fit and plausible scaling pathways. As an analytical lens, it supports comparative learning and anticipates institutional constraints when scaling NbS across heterogeneous urban contexts.

**Supplementary Information:**

The online version contains supplementary material available at https://doi.org/10.1007/s13280-026-02421-y.

## Introduction

### Background and scope of existing NbS typologies

Nature-based solutions (NbS) are increasingly recognised as a cornerstone of urban sustainability strategies that offer multifunctional and cost-effective responses to intersecting socio-ecological challenges such as climate adaptation, water management and biodiversity loss (Seddon et al. 2020). NbS are defined by their use of (semi-)natural systems to deliver ecosystem services (e.g. regulating services like hazard mitigation) and societal benefits (Pauleit et al. 2017). Their relevance has been codified through internationally recognised frameworks, including the IUCN (International Union for Conservation of Nature) Global Standard (Cohen-Shacham et al. 2016) and the EU (European Union) Biodiversity Strategy 2030 (Maes and Jacobs 2017).

As NbS have gained prominence, the need for systematic classification has intensified. Existing NbS classifications and their typologies, each with distinct organising principles (Table S1), have been central to promoting their mainstreaming by providing a common basis for how NbS are conceptualised, designed and compared across contexts (Chausson et al. 2020). For instance, challenge-based typologies (e.g. those linked to the Sustainable Development Goals) aligned NbS measures with societal priorities such as climate change, disaster risk reduction and food security (Cohen-Shacham et al. 2016). Ecosystem-based typologies (e.g. wetlands, forests) grounded measures in ecological logic to facilitate integration with ecosystem service assessments (Eggermont et al. 2015). Intervention-based (e.g. green infrastructure or ecosystem-based adaptation) and scale-based (e.g. spatial distinctions between site, neighbourhood and city, or temporal distinctions between short- and long-term) typologies also provided design orientation and helped delineate scope and functional scale (Lafortezza et al. 2018; Enu and Pauleit 2025). Thus, these existing typologies tend to focus on the various ecological processes and benefits, or their spatial and technical design form.

However, the outcomes of NbS are not determined by technical design alone but are at least equally dependent on governance fit (Kiss et al. 2019). While some frameworks acknowledge governance (e.g. challenge-based typologies may note policy actors and ecosystem-based typologies may imply land or resource control), none systematically integrates key levers shaping decision-making and long-term responsibility. In particular, existing typologies rarely address decision authority beyond the identification of actors, resource mobilisation and control beyond mere availability or the role of knowledge in conferring legitimacy and structuring stewardship. Even multidimensional typologies that combine logics (e.g. ecological with technical) rarely make governance variables explicit. This omission stems likely from the implicit assumption that technically sound interventions will succeed regardless of institutional arrangements. Yet in practice, feasibility and sustainability depend greatly on who holds authority, who mobilises resources and how coordination is maintained (Egusquiza et al. 2021). Treating governance as secondary obscures differences in actor roles, overstates the benefits of participation and masks critical variation in governance modes and implementation outcomes (Malekpour et al. 2021). It also underrepresents informal and non-state interventions, which are central to scaling NbS for urban adaptation (van der Jagt et al. 2021). These omissions reinforce an “installation bias” where NbS are treated as technical artefacts that deliver value regardless of how they are governed (Kabisch et al. 2022; Kauark-Fontes et al. 2023).

### Why governance matters for NbS classification

Governance refers to the arrangements of actors (Zingraff-Hamed et al. 2021), authority and rules (Lemos and Agrawal 2006), resources (Kiss et al. 2022), knowledge and responsibilities (Buijs et al. 2024) through which decisions shaping how interventions are initiated, coordinated and sustained are made. For cities, governance is critical because infrastructural complexity, fragmented authority and contested land ownership often lie beyond direct state control (Krawchenko and Tomaney 2023), resulting in diffuse implementation arrangements and unclear mandates (Mahmoud and Morello 2021). These conditions mirror climate adaptation challenges, where limited local capacity, institutional misalignment and jurisdictional fragmentation constrain responses (van den Berg et al. 2015), and where power and priorities shape who leads (Sovacool and Linnér 2016). This leadership question is frequently reduced to binary framings such as top-down versus bottom-up or state-led versus community-led initiatives, distinctions that are too crude to capture the diverse actor constellations shaping how NbS are delivered and scaled, as reflected in NbS databases such as the Urban Governance Atlas^1^. These obscure mixed and evolving actor constellations through which key governance levers are negotiated, as governance rarely proceeds through substitution but instead layers diverse modes (hierarchical, market, network, self-governance) over time (Driessen et al. 2012). This layering is especially visible in urban NbS, which often take form through state–market arrangements or state–community partnerships. Regardless, non-state initiatives typically operate within state regulatory frameworks, and their system relevance often depends on institutional alignment, learning and facilitation by public authorities (Buijs et al. 2016). Reviews confirm that coordination, learning, participation and legitimacy often shape NbS outcomes as strongly as technical design (van der Jagt et al. 2021; van der Jagt et al. 2023a, b).

While the environmental governance literature explains why governance matters for NbS implementation, it is less explicit about why governance must also be integral to NbS classification. Existing NbS typologies treat governance as a contextual background rather than a defining dimension, limiting their ability to explain why similar designs diverge in terms of feasibility, long-term viability, or scaling trajectories. This is visible when comparing large-scale NbS projects (grounded in state authority) with decentralised interventions (driven by non-state actors). For example, flagship projects like *Room for the River* in the Netherlands (Zevenbergen et al. 2015) and the *Isar Restoration* (Zingraff-Hamed et al. 2023) in Germany show what is possible under centralised authority, yet remain costly, complex and difficult to replicate because they require consolidated mandates, multi-level coordination and long-term public budget commitments (Albert et al. 2021a, b; Zingraff-Hamed et al. 2021). By contrast, meso- and micro-scale interventions (e.g. community gardens, green roofs) provide distributed pathways to adaptation and often rely on voluntary uptake across fragmented land ownership (Wamsler et al. 2020a). Their effectiveness and durability depend equally on governance arrangements that align key levers. Scaling such interventions requires governments to shift from direct provision towards enabling and connective roles (mosaic governance) linking diverse initiatives into broader urban green infrastructure (Buijs et al. 2019). But prevailing NbS typologies treat public agencies as the default locus of decision-making (Hübner et al. 2021) and use static categories (Morita and Matsumoto 2021).

Neglecting governance as a classificatory dimension therefore obscures variation in feasibility, sustainability and equity that is determined by who controls key governance levers. Work on wider uptake shows that scaling depends on alignment among actors, rules, resources and problem framings rather than on intervention design alone (Dorst et al. 2021). Recent advances propose governance assessment frameworks that evaluate process quality, including inclusiveness, collaboration, learning and legitimacy (van Der Jagt et al. 2023a, b). While essential, these approaches assess how well governance performs rather than distinguishing how governance is organised across interventions and, as such, do not enable typological comparison. They also specify where enabling regulation, finance and maintenance arrangements must shift when authority and responsibility remain dispersed across actors locally. A governance lens oriented towards classification extends NbS typologies beyond what interventions are and deliver, to how authority, responsibility and stewardship are organised at implementation, clarifying why similar NbS follow different pathways and what scaling requires. In practice, this governance diversity is already emerging as cities increasingly design regulatory, financial, and technical assistance tools to enable diverse actors, including citizens, to implement NbS (Fakirova et al. 2025). However, since this shift has not been reflected at the conceptual level of NbS classification, such programmes are at best reduced to coarse state/non-state-initiated distinctions.

### Study rationale and objective

The analytical gaps in NbS governance research related to classification have practical implications. These include the fact that it undermines the systemic transformations envisioned by key strategies like the EU Green Deal, the Climate-Neutral and Smart Cities Mission and the UN (United Nations) Sendai Framework, which all call for co-created solutions like NbS to move from isolated pilots to distributed mainstream infrastructure (Collier et al. 2023). Without clarity on who leads, who decides and who is accountable, NbS risk being treated as generic green infrastructure rather than interventions that actually have adaptive effects and whose impacts hinge on institutional alignment (Wamsler et al. 2017). Moreover, governance opacity obscures how responsibilities and benefits are allocated, raising equity concerns when implementation burdens fall disproportionately on actors with limited capacity (Cooper et al. 2023). These challenges emphasise the need for a classification that renders governance arrangements explicit and comparable.

With this background, the aim of this paper is to develop a governance-informed typology of NbS that classifies interventions by the lead actor to complement existing typologies that emphasise ecological design and function and to discuss its theoretical and practical implications. Even though governance arrangements are often diffuse and difficult to grasp, a leadership-centred perspective makes them analytically visible, improving how NbS are integrated into policy and scaled across contexts. The typology is derived from retrospective classification of selected NbS cases, providing a forward-looking basis for anticipating governance needs in planning and policy design. The core contribution of this paper is to extend prevailing NbS typologies by making governance a core classificatory dimension. Drawing on established governance scholarship, we operationalise existing governance concepts into an implementation leadership framework that renders governance arrangements comparable across interventions and reveals institutional strengths, vulnerabilities and scaling constraints that remain invisible in existing NbS classifications.

## Conceptual framework: A governance-informed, leadership-centred typology

### Positioning implementation leadership in governance scholarship

Generally, implementation leadership can be understood as the linking mechanism between formal governance levers (e.g. policy designs, and institutional mandates, and the situated practices) through which interventions are operationalised (Ansell and Gash 2012). Recent environmental governance scholarship has moved the conceptualisation of leadership from a fixed attribute of individuals or organisations to a distributed, relational and embedded in implementation processes (Frantzeskaki 2019; Torfing and Sørensen 2019). Hence, leadership functions as an operational governance capacity through which formal commitments are translated into sustained outcomes, particularly for complex interventions such as NbS that unfold across multiple actors and time horizons (van der Jagt et al. 2021). This framing aligns with process-oriented governance research showing that governance levers are continually (re)negotiated during implementation under conditions of uncertainty, multi-actor involvement and long-term responsibility (Driessen et al. 2012).

This shift has enabled research to examine how interventions are organised and coordinated in practice through analytical concepts focused on authority and accountability structures, stewardship and place-keeping arrangements and institutional capacity (Martin et al. 2021; Van Cauwenbergh et al. 2022). Across these perspectives, the shared insight is that implementation outcomes depend less on design quality alone than on the capacity to align stakeholders, mobilise resources (Bixler et al. 2020) and exercise leadership under conditions of institutional complexity and uncertainty (Ansell et al. 2017). In the context of NbS, leadership is thus primarily a matter of orchestration, as delivery unfolds across dispersed centres of authority and hybrid governance arrangements that require sustained collaboration and coalition-building over time (Zingraff-Hamed et al. 2021).

Despite their explanatory power, these governance concepts do not provide an intervention-level classificatory principle that renders NbS governance levers comparable across cases, a limitation that is particularly evident for non-state and hybrid initiatives whose success depends on sustained institutional and resource commitment beyond local motivation (Runhaar et al. 2018; Kabisch et al. 2022). We therefore introduce implementation leadership as an analytical anchor that captures the convergence of decision authority, enabling capacity and long-term stewardship responsibility during implementation, linking system-level governance arrangements with intervention-level outcomes.

### Governance across the NbS lifecycle and classifying at institutional stabilisation

Governance mediates NbS delivery across planning, implementation and stewardship phases (Albert et al. 2021a, b). In planning, influence is dispersed through institutional plans and procedures (e.g. mandates, standards, assessments, consultation) that define admissible problems, designs and participants; in implementation, influence becomes decisive as delivery depends on coordinated roles and enforceable control over land, finance and permits; and in stewardship, influence is revealed as maintenance and adaptation obligations encounter institutional discontinuities (e.g. budget cycles, staff turnover, shifting priorities) (Hegger et al. 2014; Nash et al. 2021). Because governance arrangements evolve across stages, early coalition formation during planning may be an unreliable indicator of long-term governance (Huitema et al. 2016). Similarly, in stewardship phases, observed maintenance outcomes primarily reflect earlier institutional commitments, while visible stewards often lack the authority or resources to alter them (Ostrom 1999; Enqvist et al. 2018). By contrast, implementation is the critical juncture at which governance arrangements become binding, as provisional commitments are converted into operative institutional control through contracts, mandates and organisational routines (Carlisle and Gruby 2019). Implementation thus bridges planning and stewardship by translating aspirational intent into institutional arrangements that enable or constrain long-term maintenance. Consequently, we classify cases at the point of institutional stabilisation, typically observable during or following implementation, when key governance levers become sufficiently settled to render leadership patterns empirically legible. Stabilisation is treated as a methodological choice rather than a claim about permanence to help avoid classifying symbolic, temporary or transitional arrangements, particularly in cases where NbS may be proposed, implemented and maintained by different actors.

### Defining implementation leadership and its justification as a classificatory anchor

Building on governance mode research, we conceptualise implementation leadership as the intervention-level locus where governance functions stabilise around specific actors during NbS delivery. We posit that implementation leadership crystallises through four levers, with each representing a distinct governance function: (i) initiation (formation), (ii) decision authority (direction), (iii) resource control (enabling capacity) and (iv) stewardship responsibility (sustained accountability).

**Fig. 1:**
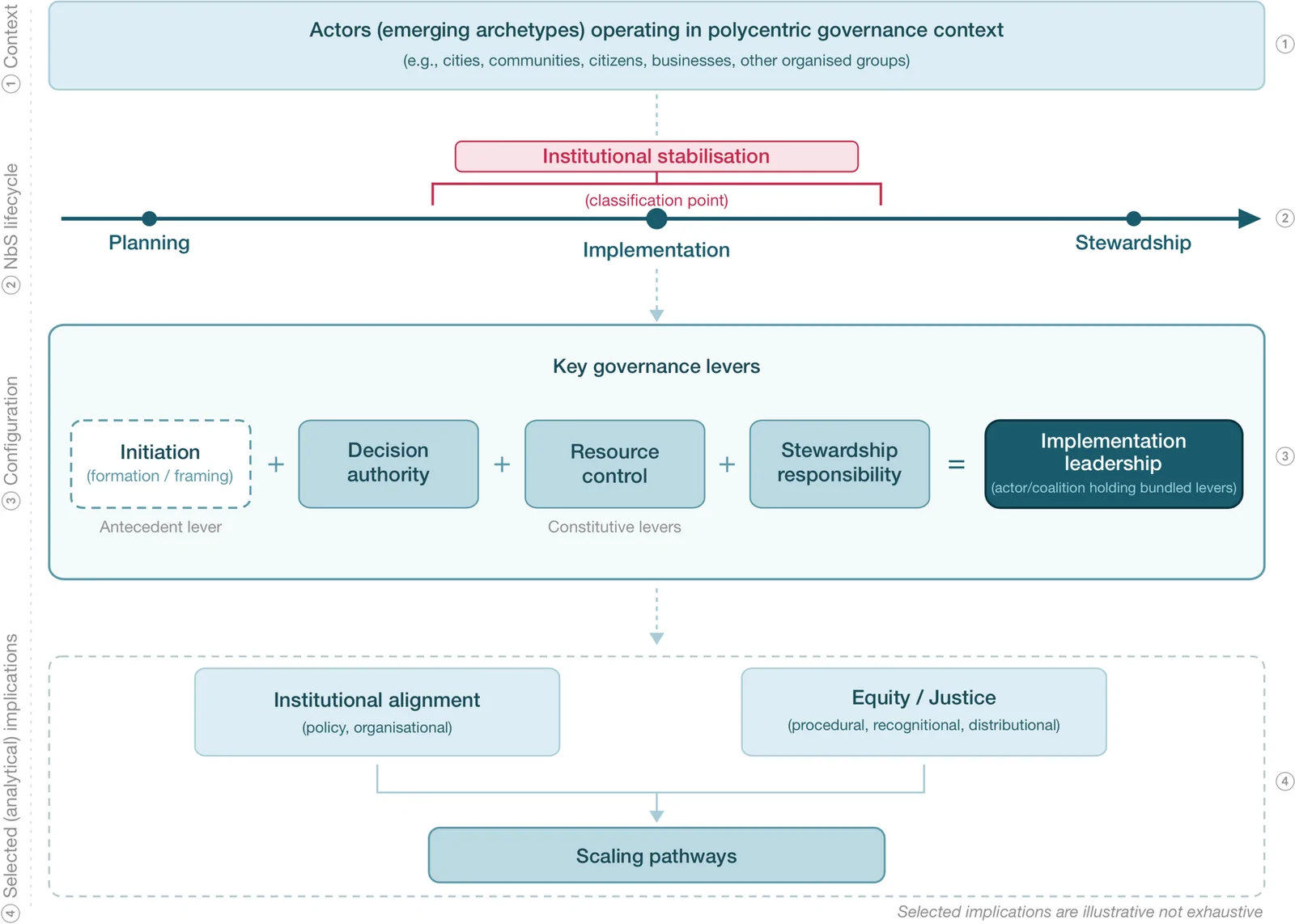
Conceptual diagram of implementation leadership with constitutive levers

We distinguish initiation as an antecedent lever and the rest as constitutive (Fig. 1). Initiation captures who originally proposed or catalysed the intervention and how nature was framed as a solution to a societal concern. For the constitutive levers, decision-making authority captures who has the strategic power to approve or amend key elements of implementation, including site selection, design, budgeting, partnership arrangements or changes to project scope (Lemos and Agrawal 2006; Kiss et al. 2019); resource control refers to which actor mobilises or regulates access to critical inputs such as land, finance, permits or technical expertise (Pahl-Wostl 2019); and stewardship responsibility identifies who assumes long-term maintenance, monitoring or adaptive governance and whether these responsibilities are institutionalised through mandates, budgets or contractual arrangements (Martin et al. 2021). Thus, at the point of institutional stabilisation, implementation leadership is defined as the actor or coalition that holds the bundled capacity to (i) exercise or broker decision authority, (ii) mobilise or control critical resources and (iii) assume responsibility for long-term stewardship.

Implementation leadership is a strong classificatory anchor for several reasons. First, it integrates the governance functions that most directly condition feasibility by capturing where authority is located, how resources are mobilised and controlled and where long-term responsibility is assigned, rather than treating these dimensions as separate descriptive variables (Cheok et al. 2020; Egusquiza et al. 2021). Second, it is observable and comparable because authority, resource and stewardship obligations are typically traceable in formal documents (e.g. mandates, programme rules, contracts, budget commitments), even when actor constellations differ (Winsvold and Vabo 2024). Third, it differentiates otherwise similar NbS interventions by revealing how implementation pathways are structured across fragmented urban systems, including where coordination burdens concentrate and which scaling routes become plausible under different leadership configurations (Malekpour et al. 2021). In this sense, implementation leadership functions as a classification-ready proxy for governance configuration at the intervention level, without collapsing diversity into a single governance mode or institutional form. Building on this definition, the “Materials and methods” section specifies evaluative questions and empirical indicators for identifying implementation leadership across selected NbS cases.

## Materials and methods

This study follows a qualitative, comparative research design to develop and empirically ground a governance-informed, implementation-leadership-centred typology of urban NbS. The design integrates conceptual deduction with inductive pattern recognition across documented cases, aiming for analytical generalisation by identifying recurring governance configurations across interventions.

### Case selection criteria and rationale

We purposively selected 35 urban NbS cases globally to capture variation in governance forms and implementation leadership while keeping the sample sufficiently bounded for detailed case-level analysis (Table S2). Our operational definition of NbS for case selection encompassed both on-the-ground ecological measures (e.g. parks, restoration projects) and regulatory or institutional instruments (e.g. mandates, incentive schemes) that directly govern or sustain such measures, to maintain empirical consistency and align with emerging scholarly definitions (Cohen-Shacham et al. 2019; Frantzeskaki and Bush 2021). Cases were identified through a structured literature search on Scopus and Web of Science (documented from 2015 to 2025, even if interventions started earlier, with the condition that governance arrangements were still in force or reshaped from 2000 onward), complemented by grey literature searches (e.g. Urban Governance Atlas, ICLEI, UN-Habitat, OPPLA) and targeted snowballing. We followed best practices in qualitative typology building by prioritising institutional contrast and conceptual saturation, understood here as the point at which additional cases no longer yielded new governance forms or archetypal patterns but only variation within existing categories (Saunders et al. 2018).

**Fig. 2:**
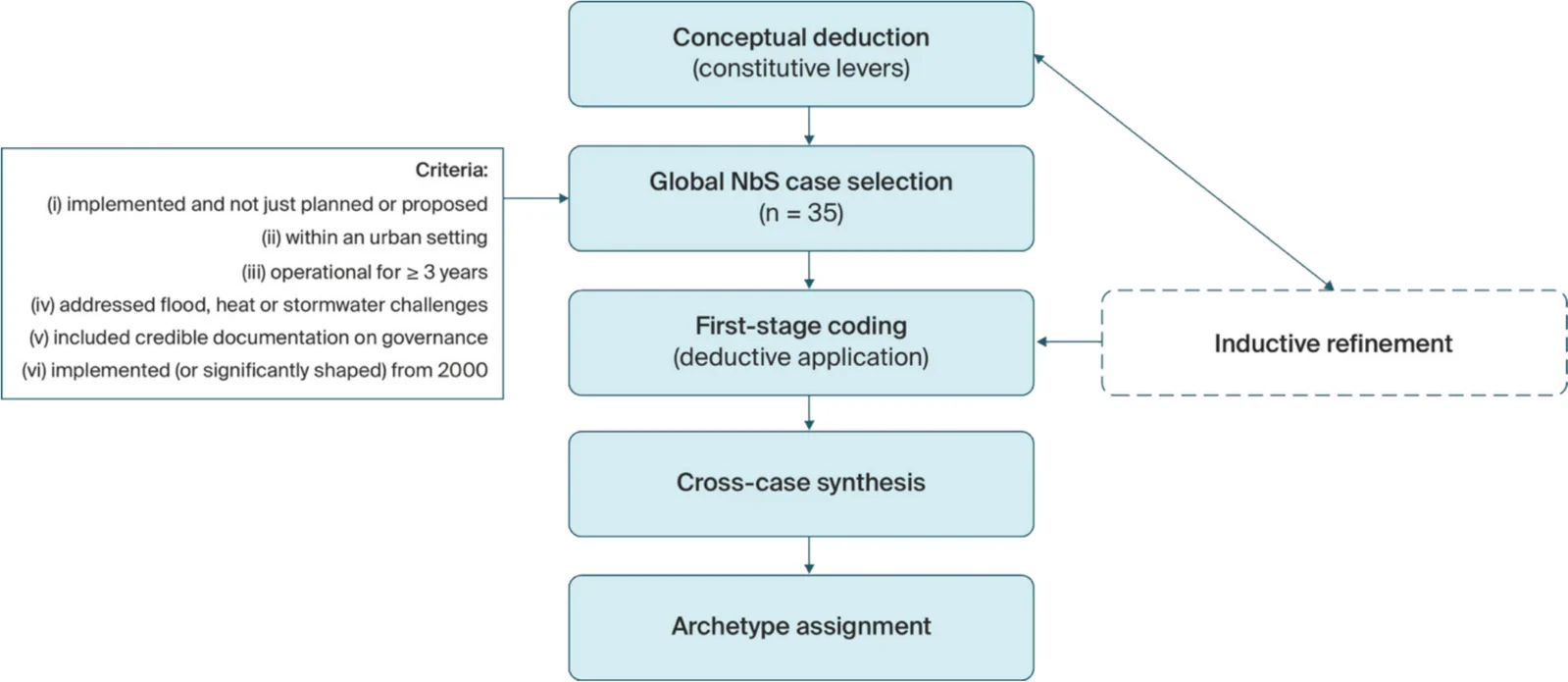
Methodological approach and workflow

Each case met six inclusion criteria (Fig. 2), including being urban, addressing common hydro-meteorological hazards relevant to climate adaptation and having been implemented and operational for ≥ 3 years to allow governance stabilisation and leadership consolidation. In applying these criteria, we found climate adaptation was often implicit rather than explicit in documentation, with adaptation-relevant interventions framed around water management, risk reduction or liveability. Given limited peer-reviewed coverage of implementation governance, we drew selectively on grey literature. To avoid discursive bias from this framing, hazard relevance was coded based on intervention function and targeted risks (e.g. flood mitigation, heat reduction), rather than the explicit use of “adaptation”. Hazard framing descriptors for each case are provided in the supplementary material (Table S2).

In line with this inclusion criteria, we applied a two-stage case selection process. In the first stage, we conducted an eligibility screening of an expanded pool of approximately 60 candidate cases. Cases were excluded if they lacked sufficient governance documentation, had not progressed beyond planning or early pilot stages or did not meet the minimum operational period required to observe stabilised leadership arrangements. In the second stage, we undertook analytical refinement towards conceptual saturation. Saturation was assessed through targeted supplementary searches to fill gaps in intervention–region combinations identified during initial coding, after which the final sample was finalised (*n* = 35). Once conceptual saturation was reached, variation rather than numerical balance guided the final sample. This reflects documented asymmetries in the institutional visibility of NbS, where macro-scale and formally codified interventions (Enu et al. 2023), largely reported from Global North contexts (Chausson et al. 2020), dominate the evidence base. Interpretation accordingly focuses primarily on recurring governance arrangements.

### Analytical procedure for operationalising implementation leadership

The four levers of implementation leadership (“Defining implementation leadership and its justification as a classificatory anchor” section) were translated into an operational coding framework (Table 1) and applied systematically across all cases. Coding focused on identifying the actor(s) responsible for each lever, the institutional form through which responsibility was expressed and whether roles were formally institutionalised or informally assumed. Because leadership is classified at the point of institutional stabilisation, coding prioritised evidence of roles becoming codified or routinised through institutional instruments (e.g. formal rules, contracts, budget lines, dedicated coordinating bodies).

**Table 1:** Operationalizing the constitutive levers of implementation leadership in NbS governance

No	Lever	Analytical focus	Guiding questions	Possible options	Relevance	References
1	Initiation and framing	Identifies the actor(s) and processes through which the NbS idea was first proposed, legitimised, and positioned as a response to a societal concern	Who initiated the intervention? How was the problem/solution framed?	Locally demanded (community/neighbourhood-driven, bottom-up mobilisation) Co-produced (jointly developed by communities and external actors, e.g. NGOs, researchers, city) Externally introduced (top-down by state, donor, private actor) Hybrid/iterative initiation (multiple initiators, evolving over time) Other/unclear	Reveals the identity of the original agenda-setter; distinguishes between bottom-up and top-down origins, which shape legitimacy and leadership evolution	Frantzeskaki (2019), Zingraff-Hamed et al. (2020), Kabisch et al. (2022)
2	Decision-making authority	Identifies who holds strategic authority to direct implementation at the point of institutional stabilisation, including approval, veto, and codification of decisions	Who approves site, design or budget? Who can veto or amend? Is authority codified?	Centralised formal authority (e.g. municipal/state agency, statutory law) Decentralised/shared authority (e.g. community boards, multi-stakeholder committees) Informal/negotiated authority (e.g. tacit agreements, customary norms, no legal codification) Hybrid authority (formal and informal coexist, negotiated across levels) Other/unclear	Identifies directive control and veto power; clarifies whether authority is institutionalised or informal, distinguishing symbolic from accountable leadership	Enqvist et al. (2018), Ostrom (2020), Egusquiza et al. (2021)
3	Resource control	Identifies who provides or regulates access to essential implementation inputs (land, finance, permits, expertise), and how these are structured	Who mobilises/controls land, funding, permits, expertise? How are resources structured?	Centralised control (concentrated in a single lead actor, e.g. state or developer) Distributed control (resources allocated across multiple independent actors) Pooled resources (co-investment, joint funding, shared expertise) Volunteer/informal contributions (labour, materials, expertise in-kind) Other/unclear	Reveals which actor controls indispensable inputs; identifies whether resource control is concentrated or shared, shaping whether governance is hierarchical, collective or negotiated	Pahl-Wostl (2019), Frantzeskaki and Bush (2021), Van Cauwenbergh et al. (2022)
4	Stewardship responsibility	Identifies who assumes long-term responsibility for maintenance, monitoring, or adaptive governance, and whether these responsibilities are institutionalised	Who maintains the intervention post-implementation? Is responsibility institutionalised in budgets, contracts, or mandates?	Institutionalised public stewardship (city/state agency budgets and mandates) Institutionalised private stewardship (developer, utility, HOA contracts) Community/collective stewardship (neighbourhood groups, NGOs) Individual/household stewardship (property owners, residents) Hybrid/shared stewardship (formal and informal actors share roles) Unclear/voluntary only (ad hoc, no budgetary/institutional guarantees)	Establishes responsibility for sustaining ecological and operational continuity; differentiates informal/voluntary from institutionalised stewardship	Wamsler et al. (2020a, b), Albert et al. (2021a, b), Martin et al. (2021)

Analysis proceeded in two stages. First, available documentation for each case was reviewed and coded against the four levers, using categories derived from institutional and governance theory and refined iteratively as empirical patterns emerged. Second, coded results were organised in a case-by-lever matrix to enable systematic comparison and identify clusters of cases with similar leadership patterns (Table S3; Miles et al. 2014). Alignment patterns across the three constitutive levers were interpreted with reference to established coordination logics in governance scholarship (hierarchical, negotiated, devolved), without treating these logics as classificatory categories in themselves. Archetypes were identified through iterative pattern recognition of how decision authority, resource control and stewardship responsibility aligned across actors, with initiation used as an antecedent indicator to trace leadership trajectories.

To ensure analytical rigour, two researchers coded cases independently and reconciled differences through joint review. Inter-coder reliability was assessed on a random subset of 100 coded text segments across constitutive governance levers; the antecedent initiation variable was excluded to avoid bias, yielding substantial agreement (Cohen’s Kappa κ = 0.80). Implementation leadership was identified retrospectively based on the stabilised patterns, which sustained delivery and stewardship. For consistent and replicable identification of leadership across heterogeneous governance contexts, we applied ex ante decision rules at the point of institutional stabilisation. Formal regulatory authority was not treated as sufficient evidence of leadership. Cases were classified as co-led where institutionalised power-sharing between public and non-state actors was evident across at least two constitutive levers, while cases with polycentric authority confined within the public sector were classified as state-led. Where sources provided conflicting accounts of authority, resource control, or stewardship responsibility, we applied a structured decision protocol, prioritising legally binding or institutionalised sources (e.g. laws, formal mandates) over descriptive or narrative sources (e.g. reports). Archetypes were assigned only where at least two of the three constitutive levers could be coded with confidence; otherwise, they were considered hybrid or transitional (Collier et al. 2012; Ansell and Torfing 2022).

### Governance-informed typological complementarity analysis

After identifying governance archetypes for each case, the cases were coded against existing NbS typological dimensions, including challenge-based, ecosystem-based, ecosystem change type, intervention modality, spatial scale and temporal horizon (Table S4). A cross-typological matrix analysis was then used to map governance archetypes across these dimensions, and the resulting patterns were visualised using an alluvial (Sankey-style) diagram to trace how identical NbS forms and challenges (mis)align with divergent leadership patterns. Analysis focused on dispersion rather than correspondence, examining whether governance archetypes clustered within or cut across prevailing NbS categories, thereby assessing governance as an independent classificatory dimension that reveals institutional variation obscured by form- and function-based NbS typologies.

## Results

This section describes the empirical breadth of the selected cases (4.1); identifies recurring archetypes (4.2); examines how the constitutive levers align at stabilisation (4.3) and when they do not (4.4); and finally shows how this governance-informed typology complements existing NbS typologies (4.5).

### Overview of selected cases

**Fig. 3:**
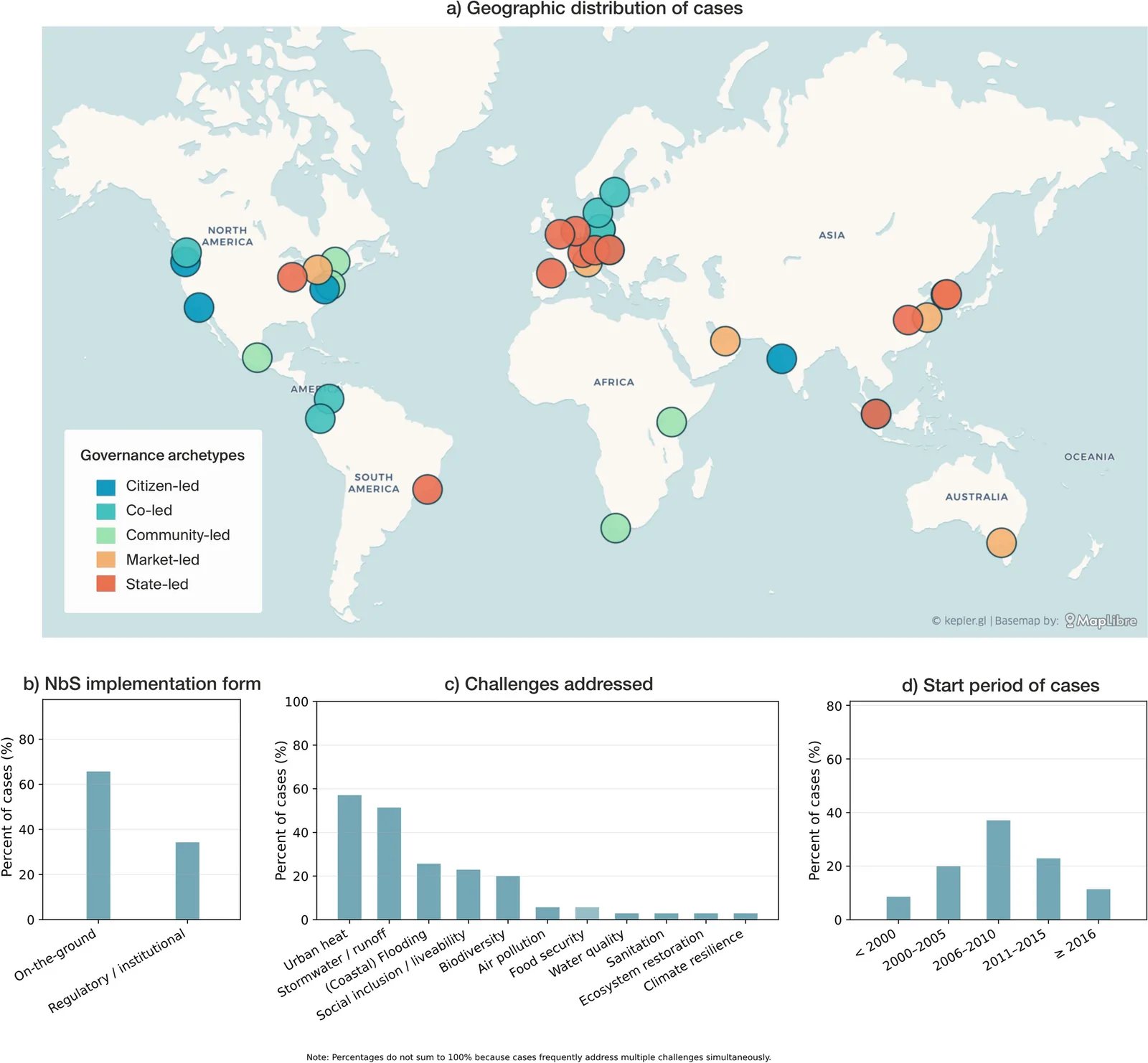
Overview of the cases. *(NB: the map is descriptive and not indicative of actual regional prevalence)*

Across the 35 selected cases, NbS implementation exhibits pronounced heterogeneity in timing, form and functional orientation, across 22 countries with at least one case in each region. Although the cases were initiated after 2000, most (*n* = 25) occurred in the post-2010 period. On-the-ground ecological measures also were predominant (*n* = 23), while over one-third of cases operated primarily through regulatory or institutional instruments (Fig. 3).

The intervention form did not align with the governance scale, as nationally coordinated programmes relied on decentralised delivery (e.g. in *Room for the River*, regional water boards and municipalities implemented centrally planned measures); locally initiated NbS frequently operated within or reshaped municipal regulatory frameworks (e.g. incentive schemes and bylaws).

Functionally, most cases commonly addressed urban heat mitigation (57%) and stormwater management (51%), often concurrently. Social inclusion and liveability (23%) and biodiversity enhancement (20%) appeared mainly as secondary or co-equal aims. Functions such as food security, air quality improvement and sanitation were comparatively rare (≤ 6%). This breadth of contexts, forms and functions provided the empirical variation needed to identify recurring archetypes.

### Emergent governance archetypes across implementation leadership

**Fig. 4:**
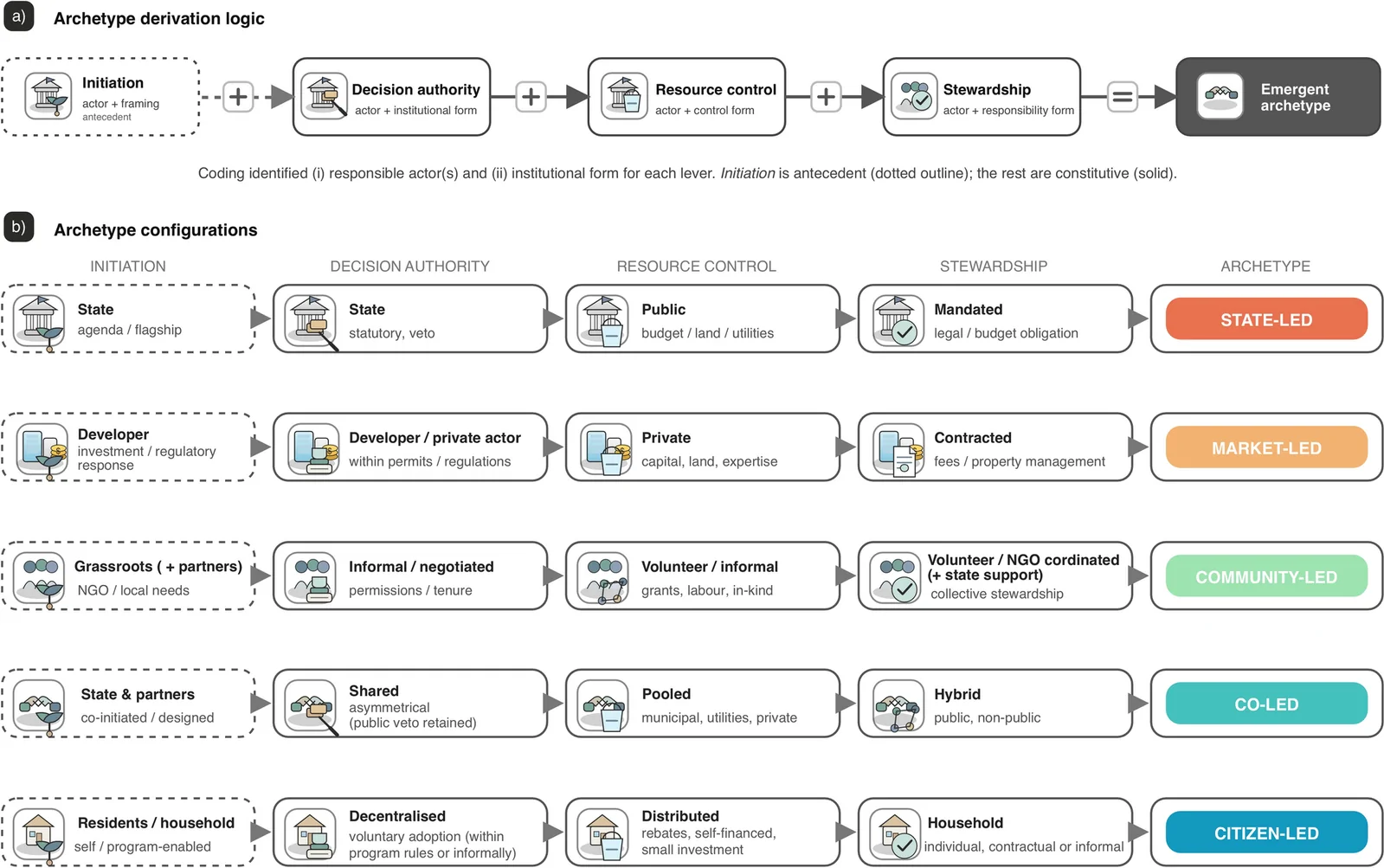
Emergent governance archetypes

**State-led:** They are illustrated by cases such as the *Basel Green Roof Mandate (Switzerland), Room for the River (Netherlands)* and *Cheonggyecheon Restoration (South Korea).* In each, leadership stabilised with statutory decision authority, control over core resources and stewardship responsibility legally consolidated within public institutions. *Basel* fixes leadership through binding building codes requiring vegetated roofs on new flat buildings and enforceable municipal permitting; *Room for the River* centralises agenda-setting, multi-billion-euro national financing and accountability within Rijkswaterstaat (Dutch Directorate-General for Public Works and Water Management) despite decentralised delivery; and *Cheonggyecheon* institutionalises leadership through municipal budgetary control and mandated public maintenance following expressway removal. Leadership thus persists because authority, resources and stewardship converge within the state, regardless of who executes implementation tasks (Fig. 4).

**Market-led:** Market-led NbS consolidate implementation leadership within private actors, aligning ecological functions with investment logics under public regulation. This is evident in *Bosco Verticale (Italy)*, where the developer (Hines) controlled finance, design and long-term maintenance through condominium contracts, with the municipality acting through its permitting authority. *Masdar City (United Arab Emirates)* and *Songdo U-Eco City (South Korea)* similarly centralised land control, capital and technical expertise within developer-led joint ventures, while states provided regulatory frameworks and infrastructure support. *Toronto’s Green Roof Bylaw (Canada)* illustrates a regulatory variant in which private developers retain design, financing and stewardship responsibilities in response to statutory requirements, thereby embedding NbS delivery within these contexts.

**Co-led:** Co-led NbS institutionalise shared implementation leadership across public and non-state actors, combining formal authority with collaborative delivery. *Copenhagen’s Klimakvarter (Denmark)* pooled municipal and utility resources under the Cloudburst Management Plan, with the city retaining statutory approval power, while residents shaped street-level design and use. In *Hammarby Sjöstad (Sweden)*, municipal agencies, utilities and private developers jointly steered planning through binding development agreements, embedding shared responsibilities in infrastructure provision and maintenance. *Berlin’s Rainwater Agency (Germany)* illustrates a softer variant, in which state agencies coordinate and provide technical guidance, while property owners decide on uptake and long-term maintenance of measures. Across these cases, decision authority is asymmetrically shared, resources are pooled, and stewardship is hybrid, situating collaboration within formal public frameworks.

**Community-led:** In community-led cases, implementation leadership stabilises where community actors, whether organised or informal, hold effective control over design decisions and ongoing stewardship, even when public guidelines or external funding are present. These cases are rooted in grassroots mobilisation and framed around locally defined socio-ecological priorities. *Montréal’s Green Alleys (Canada)* exemplify negotiated authority: resident committees initiate proposals, co-design layouts and assume maintenance responsibilities under borough guidelines, supported by modest municipal grants (approximately CAD 7000–25 000 per alley) and technical advice. *Prinzessinnengarten (Berlin, Germany)* operates through temporary land-use agreements (initially short-term leases of one to three years) with volunteer labour coordinated by a non-profit association and without long-term tenure security. In the *Kibera Public Space Project (Kenya),* community-based organisations co-manage riparian public land in partnership with KDI (Kounkuey Design Initiative), pooling donor funds while retaining day-to-day site stewardship under informal tenure conditions. Leadership therefore stabilises where decision-making authority and stewardship responsibility converge within community organisations, while external actors may provide enabling resources.

**Citizen-led:** They stabilise where households retain effective control over uptake, siting and long-term care even when initiatives are structured as public programmes. In *Philadelphia, RainCheck* and *Washington, DC’s RiverSmart Homes (United States),* municipal water agencies define technical standards and approve contractors, yet participation is voluntary, installations occur on private parcels, and homeowners sign multi-year maintenance agreements that assign upkeep responsibility for rain gardens, barrels or permeable paving to the property owner. By contrast, *Guerrilla Gardening* (*Los Angeles, United States)* and *Rooftop Gardening (Mumbai, India)* represent informal variants in which residents initiate projects on vacant lots or private rooftops, self-finance materials or rely on small collective contributions and assume maintenance without formal mandates or budgeted stewardship arrangements. In these cases, decision authority over adoption remains decentralised, resources are mobilised through dispersed household investments or targeted rebates rather than consolidated public budgets, and stewardship responsibility rests primarily with individuals, mediated by enabling but non-directive regulatory frameworks.

### Alignment of governance levers and stabilisation of implementation leadership

Across all citizen-led and neighbourhood-led cases, formal decision-making authority, expressed through regulatory approval, eligibility rules, technical standards and veto rights, remained with public actors. Leadership nevertheless stabilised outside the state, where control over implementation resources and long-term stewardship rested with non-state actors. Particularly, incentive-based programmes such as *Rain Check, Clean River Rewards (United States)* and the *Skyrise Greenery Incentive Scheme (Singapore)* illustrate this pattern as public agencies retained rule-setting, inspection and compliance functions, while households exercised discretion over uptake and design and assumed ongoing maintenance responsibilities, resulting in citizen-led stabilisation despite sustained regulatory oversight. Neighbourhood-led cases (e.g. *Montreal’s Green Alleys, Prinzessinnengarten, Kibera Public Space Project*) show a parallel pattern: municipalities control land or permits, while organised civic groups mobilise pooled resources and sustain collective stewardship, stabilising leadership outside public institutions.

**Fig. 5:**
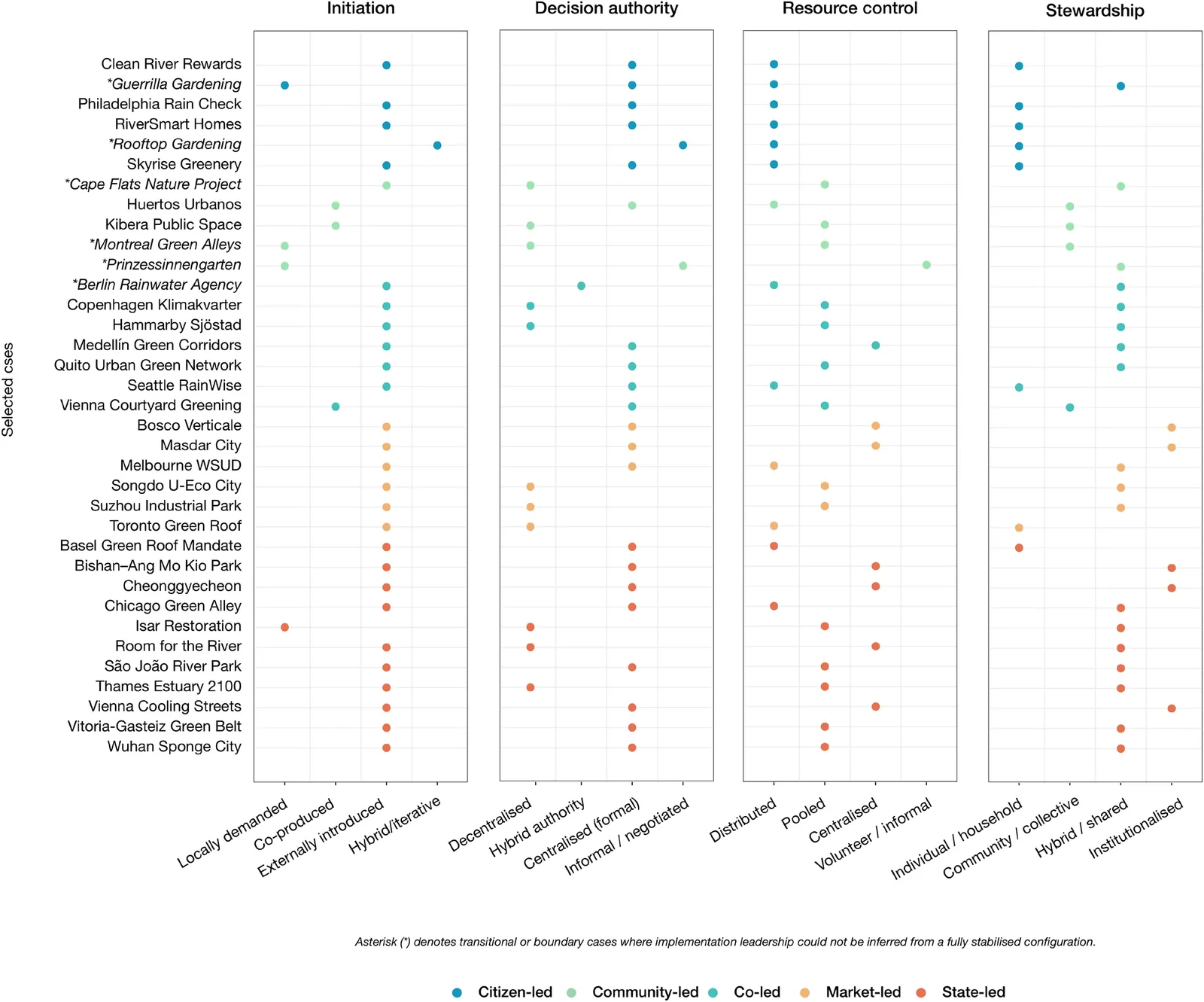
Implementation leadership configurations across NbS cases

Initiation proved empirically unreliable as a classificatory signal because most cases were externally introduced (77%), meaning that the NbS were first proposed or framed by actors, such as public authorities, utilities, developers or NGOs, other than direct local beneficiaries (Fig. 5). Several locally demanded or co-produced initiatives nevertheless stabilised as state-led once decision authority, funding streams and stewardship obligations were codified through statutory programmes or intergovernmental agreements, such as the *Isar Restoration (Germany)*, where civic mobilisation around river renaturation took strong root in the 1980s, leading to a formal collaborative planning process established in 1995 and culminating in state-led implementation under the *Isar-Plan* from 2000 to 2011. Conversely, some state-initiated programmes stabilised as citizen-led, with participation remaining voluntary and stewardship household-based, as in *Philadelphia’s Rain Check (United States)*, where the Water Department designs and subsidises installations, while participating property owners sign maintenance agreements and assume long-term upkeep of measures (e.g. rain gardens, barrels and permeable surfaces) on their private land.

### Boundary cases

Boundary cases sit at the margins of the archetypes, where implementation leadership could not be inferred from a settled arrangement because the constitutive levers remain only partially aligned or shift over time, producing hybrid or transitional governance forms. Even so, implementation leadership proved robust during institutional stabilisation, as such cases were few (17%).

Five cases exhibited transitional boundary conditions in which implementation leadership reconfigured through the (i) codification (formalisation of previously informal or contested authority or rules; e.g. *Guerrilla Gardening (United States)*, where ad hoc planting practices later intersected with municipal enforcement and negotiated toleration), (ii) consolidation (anchoring of decision authority, resource control and stewardship within statutory mandates and budgeted programmes; e.g. *Montréal’s Green Alleys (Canada)*, which evolved from neighbourhood initiatives into municipally supported and funded programmes) or (iii) withdrawal (loss of institutionalised resources and programme support; e.g. *Cape Flats Nature Project (South Africa)*, where donor reliance and unstable public backing limited long-term consolidation) of constitutive levers during implementation (Table 2).

**Table 2:** Boundary cases and forms of governance instability

Case	Boundary condition	Constitutive lever(s) affected	How alignment is disrupted
Cape Flats Nature Project, South Africa	Transitional-withdrawal	Resource control; Stewardship	Donor-funded resources and programme coordination were withdrawn, leaving stewardship non-institutionalised and responsibility fragmented
Guerrilla Gardening, USA	Transitional-codification	Decision authority	Initially informal and contested practice later became formally permitted, shifting authority without parallel institutionalisation of resources or stewardship
Prinzessinnengarten, Germany	Transitional-incomplete consolidation	Decision authority; Stewardship	Temporary land-use contracts and reliance on voluntary labour prevent consolidation of authority and long-term responsibility
Rooftop Gardening—Fresh & Local, India	Transitional-iterative/informal	Decision authority; Stewardship	Hybrid initiation persists without consolidation of authority or institutionalised stewardship beyond household scale
Montreal Green Alleys (Ruelles Vertes), Canada	Transitional-consolidation	Decision authority; Resource control	Bottom-up community initiative later anchored in municipal programmes, while stewardship remains community-based, resulting in partial but incomplete alignment
Berlin Rainwater Agency (Regenwasseragentur), Germany	Hybrid-persistent split	Decision authority; Stewardship	Advisory coordination is institutionalised without transfer of approval authority or stewardship responsibility, producing a stable but non-convergent configuration

By contrast, *Berlin’s Rainwater Agency (Germany)* represents a hybrid boundary case in which advisory coordination was institutionalised through a dedicated agency structure, yet approval authority, budgetary control and long-term stewardship remained distributed across municipal departments and property owners, resulting in a persistent split configuration rather than convergence towards a single leadership centre.

### Governance-informed classification in relation to existing NbS typologies

Applying the governance archetypes showed that interventions clustered together in prevailing NbS typologies diverge sharply once implementation leadership is considered, with no typological category mapping consistently onto a single governance archetype or any distinctive ecological form, challenge framing, spatial scale or temporal horizon. This pattern is most evident within the intervention modality of green infrastructure, the dominant technical orientation in the sample (74%), which encompasses diverse measures and spans all five governance archetypes, with no archetype accounting for > 23% of cases. Despite high technical standardisation and similar functional intent, governance diversity is maximal. Green roofs illustrate this divergence, as this identical intervention form is governed by state-led mandates (*Basel*), market-led compliance and incentive regimes (*Toronto*) and voluntary household uptake with citizen stewardship (*Washington DC*) across contexts.

**Fig. 6:**
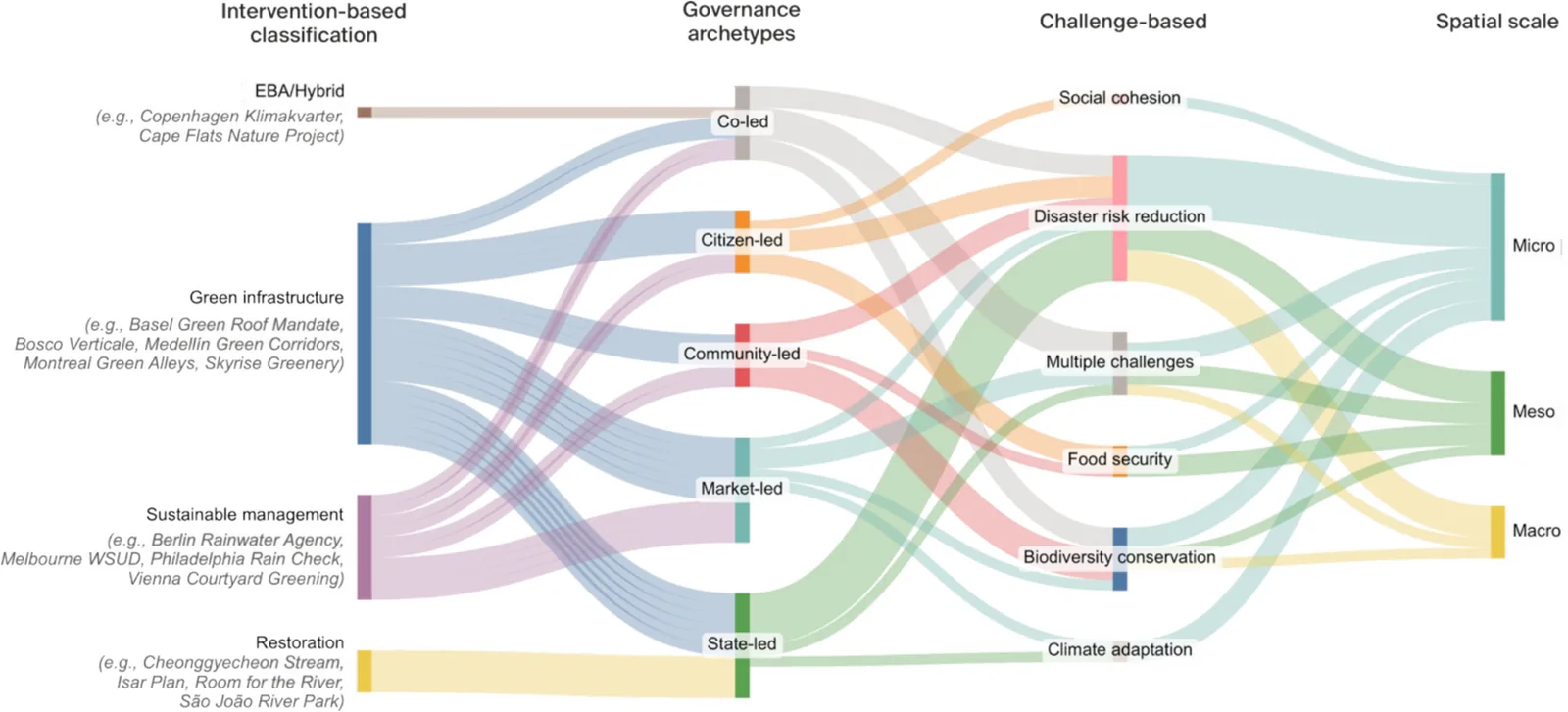
Alignment of emerging governance archetypes with (some existing) existing NbS classifications (see Table S4 for more details). (The figure highlights how governance configuration mediates the translation of similar interventions into varied outcomes). Together they enable a governance-sensitive scaling assessment linking design coherence with institutional structure. For details on existing NbS typologies, see Table S1)

Comparable dispersion appears within challenge-based typologies (Fig. 6). Although several of the cases are disaster risk reduction and water management, these interventions range from statutory state programmes (*Room for the River, Netherlands; Thames Estuary 2100, United Kingdom*) to market-led regulatory compliance (*Toronto Green Roof Bylaw, Canada*), co-led partnerships with pooled authority (*Copenhagen Klimakvarter, Denmark; Seattle RainWise, United States*) and citizen-led incentive models dependent on household maintenance (*Clean River Rewards, United States*). Shared hazard orientation, therefore, does not predetermine governance arrangements.

Ecosystem- and scale-based (spatial and temporal) typologies similarly under-specify governance. Urban terrestrial NbS cases (71%) encompassed all archetypes, and even where “ecosystem type” and “scale” coincide, governance varies substantially in how authority and stewardship are institutionalised. For example, large-scale river restoration ranges from nationally coordinated programmes with decentralised discretion (*Room for the River, Netherlands*), to mayoral flagship projects with dedicated management structures (*Cheonggyecheon, South Korea*), to consolidation following civic mobilisation through jointly formalised agreements (*Isar Restoration, Germany*). Micro- and meso-scale interventions occur across all archetypes, demonstrating that smaller scale does not imply civic leadership, nor does larger scale guarantee uniform state control. Notably, governance diversity is greatest in typological clusters characterised by high technical replicability, particularly micro-scale green infrastructure, indicating that technical uniformity does not constrain institutional variation but enables multiple implementation pathways.

## DISCUSSION

### Theoretical and practical implications

The following discussion advances theoretical and practical implications by clarifying implementation leadership as a stabilising governance mechanism (5.1) and classificatory lens (5.2), and by showing how it supports targeted policy alignment (5.3), justice and equity anticipation (5.4), correction of decentralised misfits (5.5) and governance-specific scaling strategies (5.6).

### Governance levers and the stabilisation of implementation leadership

Implementation leadership stabilises when decision authority, resource control and stewardship responsibility converge in a single governance arrangement. Across the 35 cases, leadership did not become identifiable through actor labels or linear shifts from state to non-state control, but when decision-making, financing and long-term maintenance were institutionally aligned. In *Basel’s Green Roof Mandate (Switzerland)*, authority, funding and regulatory enforcement converged within municipal structures, stabilising state leadership. By contrast, in *RiverSmart Homes (United States)*, public agencies set standards and provide rebates, yet long-term stewardship and uptake discretion rest with property owners, stabilising citizen-led implementation. Similarly, community-led cases, such as *Montréal’s Green Alleys (Canada),* consolidate leadership with neighbourhood groups controlling project design and maintenance even when municipal support is present. Leadership is therefore most analytically visible at the point of institutional consolidation rather than at initiation.

This stabilisation logic also clarifies why the mere presence of public actors cannot be taken as evidence of state leadership. Nearly all cases involved public rule-setting, standards or veto rights, yet these often coexisted with decentralised control over resources and stewardship. This reflects governance layering rather than substitution (Runhaar et al. 2018; Van Cauwenbergh et al. 2022), consistent with adaptation governance findings that public institutions frequently retain oversight, while implementation authority is distributed (Hegger et al. 2014). In *Songdo U-Eco City (South Korea)*, for example, regulatory frameworks remain public, but capital mobilisation and operational control are market-led. Co-led arrangements, such as *Copenhagen’s Klimakvarter (Denmark),* distributed authority and pooled resources across municipal and civic actors. However, stabilisation may shift leadership towards the state when decision-making authority, funding streams and long-term stewardship are formally consolidated within statutory mandates, even if initiatives originated locally, as in the *Isar Restoration (Germany)*. Conversely, externally initiated schemes such as *Clean River Rewards* and *RiverSmart Homes (United States)* stabilised as citizen-led because participation and maintenance remained decentralised under enabling regulatory frameworks. Thus, leadership lies not in who regulates (or initiates), but in who ultimately pays, implements and maintains.

Some cases, such as *Berlin’s Rainwater Agency (Germany)* and *Guerrilla Gardening (Los Angeles, United States)*, illustrate hybrid and transitional governance forms that blur archetypes and show how civic action can evolve into more institutionalised arrangements. These trajectories confirm the governance theory insight that institutional arrangements are dynamic and renegotiated through activism, learning and power-sharing (Driessen et al. 2012). Recent studies further confirm that governance shifts matter not only for which forms emerge, but also for how they work, since power asymmetries and authority-sharing shape whether collaboration succeeds or stalls (van der Jagt et al. 2021). The dynamic trajectories observed across the 35 cases empirically validate the focus on institutional stabilisation as the analytically robust basis for classification, in that it captures the moment when accountability, capacity and responsibility consolidate in a way that enables comparison and prospective planning (Huitema et al. 2016). Surfacing these dynamics ensures that the governance-informed classification provides a more realistic basis for analysing how interventions evolve, consolidate or shift roles over time, and equips policymakers with the analytical basis to anticipate when interventions may require new forms of support, authority-sharing or institutional anchoring. Fundamentally, shifting attention from origin narratives to where governance levers converge renders governance analytically visible at the intervention level and moves beyond binary state/non-state coding.

### Governance arrangements explain variations in implementation pathways and effectiveness that are invisible in existing NbS typologies

Applying the governance-informed classification across the selected NbS cases revealed systematic variation in implementation leadership and in the distribution of constitutive governance levers (authority, resource control and stewardship responsibility) that existing typologies (Table S1) do not capture. Interventions that appear similar in terms of technical design or ecological function diverge significantly when institutional structures are examined. This is particularly evident in cases that function less as discrete projects than as governance instruments, such as mandates (e.g. *Basel Green Roof Mandate, Switzerland*), incentive schemes (e.g. *Toronto Green Roof Bylaw & Eco-Roof Incentive, Canada*) and planning guidelines (e.g. *Melbourne WSUD Guidelines, Australia*), which enable distributed implementation under different leadership forms. The comparison of green roofs in *Toronto, Basel* and *Washington D.C.*, implemented under market-led, state-led and citizen-led configurations, respectively, illustrates how governance, rather than ecological form, accounts for this divergence, shaping entry points for policy instruments, the feasibility of coordination and conditions for sustained stewardship (Martin et al. 2021).

At the same time, this analysis does not render ecologically or design-based typologies redundant. Ecological classifications remain essential for identifying the ecological functions and technical principles, including what ecosystems and processes are mobilised and how design choices affect performance. Rather, the governance-informed typology complements these by making visible the institutional architectures through which such ecological functions can be realised or constrained. Importantly, the lenses are interdependent, as ecologically robust designs require enabling governance to be realised (Kiss et al. 2019), while well-aligned governance supports continuity, stewardship and learning that preserve ecological functionality (Frantzeskaki and Bush 2021). This interdependence also speaks to the point raised in NbS governance scholarship that the quality of governance processes, including inclusiveness, collaboration and legitimacy, shapes ecological outcomes as strongly as technical design (van der Jagt et al. 2023a, b). When governance arrangements are poorly matched to local authority and capacity, NbS often fail, as shown by green infrastructure pilots in Mexico City (Garcia Ferrari et al. 2022) and community water retention schemes in Cape Town (Ziervogel et al. 2016), where responsibility was devolved without commensurate authority or resources. Conversely, governance forms that integrate ecological design within appropriate governance structures (e.g. co-led arrangements linking planners and ecologists) tend to maintain functionality and resilience over time (van Der Jagt et al. 2023a, b). Even though diversity exists within respective archetypes, clustering remains useful because interventions led by the same type of actor tend to rely on similar governance tools. This reframing enables planners, funders and researchers to move beyond generic prescriptions towards governance-specific strategies that anticipate misalignments between actor mandates and implementation demands and to design tailored supports such as delegated authority, fiscal instruments or facilitation mechanisms (Table 3).

**Table 3:** Matching NbS governance archetypes with support strategies derived from dominant implementation leadership levers. NB: Interests and capacities are informed by institutional and governance theory (Scott 2013; Frantzeskaki 2019; Pahl-Wostl 2019). Support strategies and scaling pathways are synthesised from policy-mix and NbS governance studies (e.g. Fastenrath et al. 2020; Malekpour et al. 2021; Nash et al. 2021; Wolff et al. 2023). Vulnerabilities are derived from comparative case analysis and adaptation governance research (e.g. Sovacool and Linnér 2016; Kabisch et al. 2022)

Governance archetype	Typical interests of lead actor(s)	Common capacities	Relevant support strategies/policy instruments	Scaling pathway tendency	Leadership-specific vulnerabilities
State-led	Regulatory compliance, public service delivery, risk reduction, political legitimacy	Statutory authority, stable public funding, technical staff, integration into planning	Legislative mandates, long-term budget lines, interagency coordination bodies, statutory monitoring frameworks	Codification (scaling through embedding in laws, policies and formal programmes)	Political turnover, budget reallocation, bureaucratic inertia
Market-led	Return on investment, brand value, regulatory compliance, client satisfaction	Capital mobilisation, design/engineering expertise, delivery capacity	Incentives (tax breaks, density bonuses), performance-based standards, PPPs, sustainability certifications	Codification (or selective replication scaling within profitable or regulatory niches)	Market downturns, short-term profit focus, inequitable benefit distribution
Community-led	Community benefit, place-making, environmental justice, local resilience	Social capital, local legitimacy, volunteer networks, small-grant fundraising	Micro-grants, land-use agreements, co-management contracts, capacity-building, recognition schemes	Diffusion (scaling via peer networks and community-to-community learning)	Volunteer fatigue, insecure land tenure, funding instability
Citizen-led	Property improvement, cost savings, environmental stewardship, amenity value	Control over private space, willingness to invest personal resources	Rebates/subsidies, simplified permitting, technical guidance, demonstration projects	Diffusion (scaling through household adoption and social modelling)	Low uptake without incentives, maintenance inconsistency, inequitable access
Co-led	Balancing public benefit and local needs, collaborative problem-solving, shared governance innovation	Blended authority, pooled resources, cross-sectoral networks	Partnership agreements, joint funding pools, intermediaries, collaborative governance platforms, adaptive co-management	Translation (scaling by informing broader policy through collaborative pilots)	Coordination drift, high transaction costs, dependency on facilitation

### Governance-informed classification enables targeted policy alignment and scaling

The governance-informed typology offers a practical way to align policy instruments with on-the-ground governance configurations. Because each archetype embeds distinct combinations of implementation leadership levers, instruments that succeed in one mode may fail in another, which impacts the performance of interventions. For example, subsidies that stimulate uptake in citizen-led programmes have little traction in market-led models where regulatory compliance is the main driver, while legislative mandates that stabilise state-led programmes can overwhelm neighbourhood initiatives. Moreover, instrument bundles must evolve over time, shifting from catalytic support during initiation to procedural integration at stabilisation. Cases such as *RainWise (United States), Vienna’s Courtyard Greening Program (Austria)* and *Hammarby Sjöstad (Sweden)* illustrate how sequencing of instruments follows governance maturity; co-financing can trigger initiation, but codification or contracts more reliably secure long-term stewardship, a pattern consistent with empirical studies in NbS governance (e.g. Nash et al. 2021). As such, the study results highlight how instruments plausibly shift across the NbS lifecycle, so that one-off incentives are eventually succeeded by contracts, budget lines and codified responsibilities as stewardship anchors (Pahl-Wostl 2019).

This perspective on instrument alignment across the NbS lifecycle also brings informal and small-scale initiatives into view (e.g. household retrofits, community-managed green spaces and civic-led micro-interventions) that are often marginalised in formal urban nature strategies despite constituting a substantial share of implementation and maintenance effort (Wamsler et al. 2020a). For these initiatives, feasibility depends less on large capital flows than on enabling regulatory conditions, simplified permitting and institutionalised stewardship arrangements that secure continuity over time (Van Cauwenbergh et al. 2022; Hölscher et al. 2023). The value of linking policy instruments to governance archetypes lies precisely in their leadership dimensions, including who frames the intervention, controls resources, holds decision authority and carries stewardship responsibility, since these determine which instruments are viable and when they are likely to be effective (Buijs et al. 2019).

Furthermore, without systematically capturing these governance arrangements, instrument choice and sequencing remain largely ad hoc, as policymakers lack visibility over where leadership levers are aligned or misaligned. Operationalising the typology through monitoring systems and datasets that record metadata on lead actors, authority structures, resource control and stewardship arrangements would transform typologies beyond descriptive taxonomies. This would make institutional conditions easier to compare and support adaptive management across NbS portfolios by helping to identify implementation frictions earlier.

### Leadership form shapes exposure to justice and equity risks

Based on observed patterns across the 35 cases, governance configuration shapes justice and equity through institutional processes that define who participates in decision-making (procedural), how benefits and burdens are allocated (distributional), and which actors, knowledges and claims are recognised as legitimate within mandates, eligibility criteria and stewardship arrangements (recognitional). Importantly, these justice exposures are not incidental outcomes but are structurally patterned by how authority, resources and responsibility are organized at the point of institutional stabilisation.

Market-led NbS (e.g. *Bosco Verticale (Italy); Suzhou Industrial Park (China)*) integrated NbS into privately controlled developments, where access to ecological benefits and influence over design were structurally tied to investment capacity, thereby concentrating advantages among property owners and higher-income residents. Co-led initiatives (e.g. *Medellín’s Green Corridors (Colombia); Berlin’s Regenwasseragentur (Germany))* enabled broader access and shared oversight, yet their collaborative arrangements also risked uneven influence within partnerships and diffuse accountability where authority was asymmetrically distributed. Community-led NbS often carried high local legitimacy and place-based recognition. Still, they struggled with insecure tenure, unstable funding and limited statutory anchoring, constraining their capacity to deliver durable, equitable long-term benefits. Citizen-led NbS exposed sharper distributional divides, as *Rooftop Gardening (Mumbai, India)* relied on resident collectives and uneven neighbourhood mobilisation. At the same time, *Washington, DC’s RiverSmart Homes (United States)* tied participation and subsidy access to property ownership, systematically excluding renters and households lacking upfront capacity. Across these, recognitional gaps were most visible where eligibility, voice and stewardship rights were structured around formal property status or state-recognised organisations, limiting the inclusion of renters, informal residents or community claims despite their exposure to implementation burdens. These patterns confirm insights from political ecology and urban justice scholarship, asserting that equity in NbS depends not only on distributional outcomes but also on how authority, resources and responsibility are structured at stabilisation (Anguelovski et al. 2018; Cooper et al. 2023).

The governance-informed typology extends this debate by showing how different dimensions of equity are structurally embedded in leadership forms from the outset, whether through statutory mandates in state-led models, property rights in citizen-led schemes, donor dependence in community-led interventions and brokerage capacity in co-led arrangements. Rather than evaluating equity solely in terms of outcomes, the typology enables anticipatory identification of justice risks associated with particular leadership  types. Recognising how such arrangements embed inequities demonstrates that achieving equity in NbS requires more than design improvements or benefit redistribution. It depends on institutional reforms that broaden authority, stabilise resource access and strengthen accountability across governance arrangements. In practical terms, this implies that state-led models must guard against technocratic closure and narrow recognitional criteria; market-led schemes require redistributive safeguards to counter capital-linked access; citizen-led approaches need mechanisms to include renters and non-owners; community-led interventions benefit from stabilised funding and formal recognition; and co-led arrangements must clarify shared accountability to prevent diffusion of responsibility. This reinforces work on mosaic governance (Buijs et al. 2019) and wide-uptake frameworks (Dorst et al. 2021) that show how justice and equity conditions are embedded in the governance architecture, enabling distributed implementation.

### Decentralising NbS without institutional backing produces structural misfits

The cross-case analysis shows that interventions consolidated through statutory mandates or formal partnerships were embedded in long-term budgets and institutional mandates. In contrast, fragile ones relying on voluntarism or informal arrangements showed greater contingency in continuity. Community-led NbS (e.g. *Cape Flats Nature Project, South Africa)* advanced through donor cycles, micro-grants and volunteer labour. At the same time, citizen-led NbS (e.g. *Clean River Rewards in the United States)* relied on individual property owners through incentive schemes or civic activism. These models demonstrated strong commitment but lacked institutional anchoring, since authority over key implementation levers (finance, land, regulatory approvals) remained concentrated in state or private actors. Across the 35 cases, this pattern consistently distinguished those that achieved stable implementation from those that remained institutionally fragile. This reflects a broader structural misfit, as financing, permitting, and monitoring systems are typically designed for utilities and city departments rather than for decentralised leadership (Wamsler et al. 2020b).

Addressing this misalignment would require more than stable co-financing; it would call for simplified permitting, participatory monitoring, and regulatory frameworks that formally recognise decentralised stewardship (Malekpour et al. 2021). Research on climate adaptation governance further confirms that unclear mandates, limited capacity and entrenched administrative silos systematically constrain local action (Sovacool and Linnér 2016). The structural misfit observed across the cases also confirms a point stressed in mosaic governance theory that unless municipalities and higher-level institutions shift from direct provision to enabling and supporting roles, decentralised NbS will remain institutionally fragile (Buijs et al. 2019). Embedding this institutional shift in policy (e.g. through simplified regulation, multi-year community grants, formalised stewardship arrangements and participatory monitoring) would help stabilise community and citizen leadership, secure continuity and enable more equitable scaling. It also points to conditions under which decentralised governance systems can develop scaling capacity, especially when institutional support is explicitly designed to fill the lever gaps that volunteer or household stewardship cannot sustain alone (Dorst et al. 2021).

### Scaling pathways are governance-specific more than intervention form-specific

**Fig. 7:**
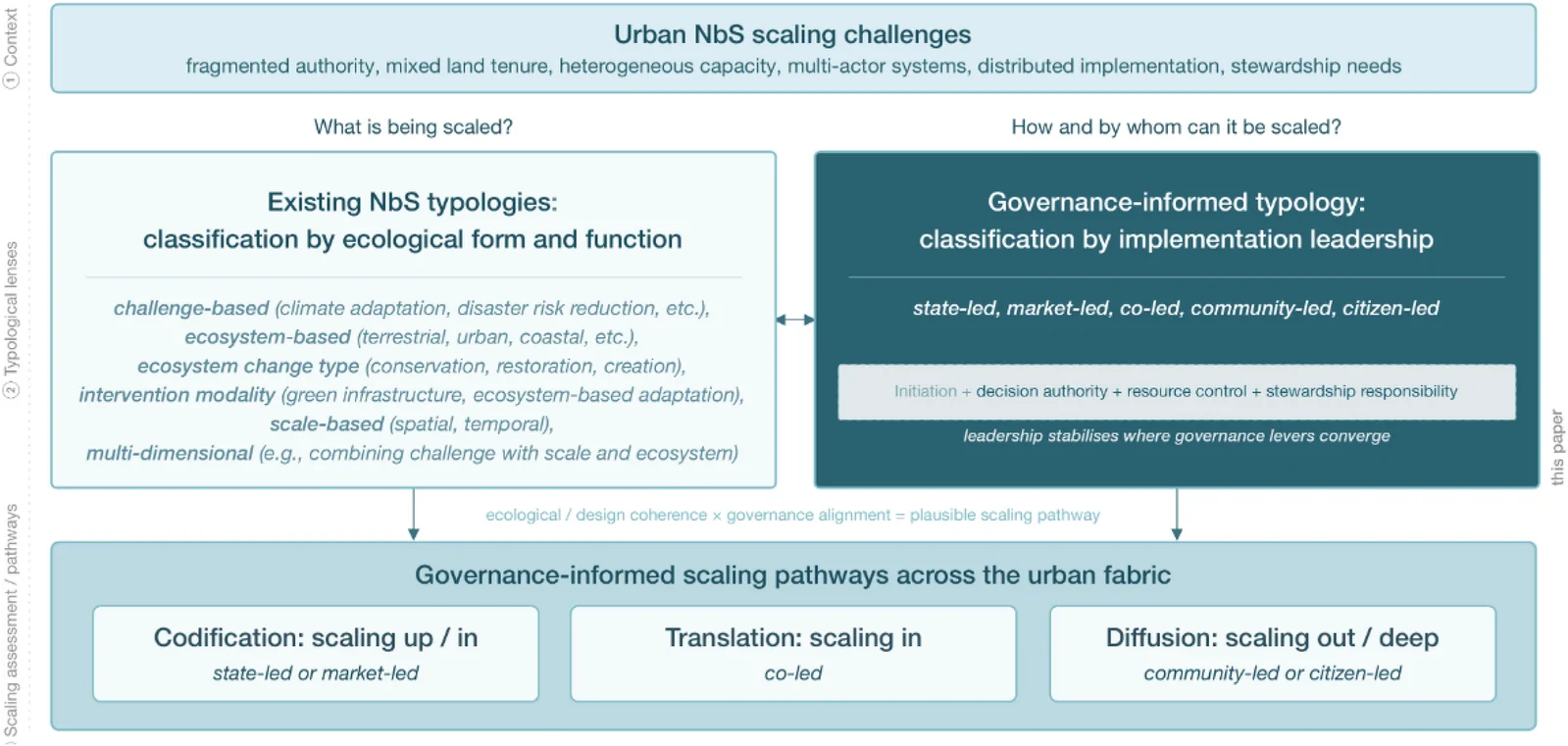
Governance-informed typology as a complementary classificatory lens for scaling urban NbS. (Existing NbS typologies classify interventions by ecological form and function; the governance-informed typology classifies by implementation leadership. Together they enable a governance-sensitive scaling assessment linking design coherence with institutional structure. For details on existing NbS typologies, see Table S1)

Despite rapid mainstreaming of NbS in policy discourse—now referenced in the national adaptation plans of over two-thirds of countries globally (Seddon et al. 2020)—empirical evidence of sustained on-the-ground scaling remains uneven, as many initiatives stall at pilot or demonstration stages due to mainly institutional and governance constraints. Although successful outcomes can support wider uptake, the pathways through which NbS consolidate, diffuse or scale are shaped more by governance arrangement and institutional structure than by intervention form alone (Fastenrath et al. 2020; Nash et al. 2021). In this sense, scaling comprises governance-dependent pathways. Citizen- and community-led forms tend to scale through diffusion and replication across households and peer networks (scaling out, often reinforced through scaling deep), while state-led forms scale primarily through codification into statutory programmes, regulatory frameworks and budgetary regimes (scaling up and scaling in) (Fig. 7). Co-led configurations operate mainly through translational pathways in which experimental partnerships reshape governance practices and inform broader policy via intermediaries and formalised accountability (predominantly scaling in, with conditional scaling up) (Midgley et al. 2021; Kabisch et al. 2022; Sarkki et al. 2024). These scaling trajectories align with insights from the policy-mix and NbS scaling literature, suggesting that governance arrangement determines institutional feasibility.

These trajectories are not explained by the intervention form. Green roofs, river restorations and urban agriculture each follow different scaling pathways depending on whether they are embedded in state mandates, civic networks or hybrid partnerships. State-led NbS, such as the *Wuhan Sponge City Program (China),* are scaled through codification, embedding into statutory frameworks and long-term budget regimes (Fastenrath et al. 2020). Community-led NbS such as *Montréal’s Green Alleys (Canada)* spread through diffusion, drawing on peer networks and informal policy emulation (Mahmoud and Morello 2021). Co-led cases such as *Copenhagen’s Klimakvarter (Denmark)* advance through translational scaling, where experiments inform broader policy through intermediaries and learning platforms. The *Isar Restoration (Germany)* illustrates how scaling pathways can shift when leadership changes, moving from civic mobilisation towards state-led codification as institutionalisation progresses. These three modes of scaling—codification, diffusion and translation—reflect distinct institutional conditions and actor constellations (Midgley et al. 2021). The governance-informed classification, therefore, clarifies which scaling mode is institutionally feasible given leadership structures, resource dependencies and institutional maturity. It also clarifies where scaling stalls (if/when authority, resources or stewardship are misaligned), enabling policymakers to align support instruments (regulatory integration, multi-year funding or formalised stewardship arrangements) with the dominant governance bottleneck rather than relying on generic scaling strategies.

## Limitations and future research directions

First, this paper does not assess the effectiveness of NbS. Instead, it clarifies how governance levers are organised at the point of institutional stabilisation, positioning implementation leadership as an analytical lens. Future research could integrate leadership-based governance archetypes with ecological monitoring and ecosystem service assessment to test how institutionalised stewardship, co-led arrangements and market-led configurations shape long-term functionality, adaptive learning and the balance between aesthetic and functional outcomes under different regulatory conditions.

Second, as demonstrated, implementation leadership provides a robust proxy for governance arrangement, but classification into archetypes necessarily abstracts from the complexity of institutional arrangements. This abstraction may obscure coordination politics in multi-actor settings where authority, resources and responsibility are distributed, contested or exercised through informal and networked influence, particularly in peri-urban or Global South contexts. The archetypes, therefore, capture dominant governance patterns and not exhaustive or universal forms. Moreover, the typology did not assess the degree, quality or inclusiveness of participation or co-design. Since cases were coded at the point of stabilisation and selected using a ≥ 3-year maturity criterion, subsequent dynamics such as institutional drift, elite capture or reallocation of stewardship responsibility fall outside the analytical frame, and early-stage or informal NbS initiatives may be underrepresented. Addressing these limitations requires longitudinal research designs that trace the evolution of governance across the NbS lifecycle in the future.

Finally, while the typology identifies who leads implementation, it does not assess leadership quality in normative terms such as legitimacy, inclusiveness or capacity. Nor does it examine how well leadership practices shape distributive, recognitional or procedural outcomes. Future research should systematically test whether distinct governance archetypes are associated with patterned procedural, recognitional and distributional justice exposures across larger samples and over time, examining whether configurations of authority, resource control and stewardship consistently predispose NbS to specific equity risks. Therefore, future NbS governance research can combine leadership-based typologies with qualitative and comparative approaches to examine how leadership is enacted, contested and perceived across contexts. Integrating such analysis with social evaluation tools would enable examination of how leadership patterns influence learning, accountability and equity, and how these dynamics, in turn, reinforce or destabilise governance arrangements.

## Conclusion

This paper develops a governance-informed typology of urban NbS by anchoring classification in implementation leadership, defined as the actor or coalition in which key levers (decision authority, resource control and stewardship responsibility) converge at the point of institutional stabilisation. In doing so, it addresses a persistent limitation of prevailing NbS typologies that prioritise ecological form, functional intent or scale while leaving governance implicit, thereby obscuring why technically similar interventions differ in feasibility, persistence, equity exposure and scaling pathways. Across 35 NbS cases, the analysis identifies five recurring archetypes (state-led, market-led, co-led, community-led and citizen-led) whose differentiation rests not on intervention type or challenge framing but on the institutional alignment of constitutive governance levers. The results show that public rule-setting is nearly ubiquitous but does not determine leadership. That initiation (as an antecedent lever) is an empirically unreliable signal because leadership commonly shifts as responsibilities are codified through mandates, contracts, programmes and budgetary routines.

The typology’s contribution lies in rendering governance arrangements comparable at the intervention level and in translating governance theory into a classification-ready lens that complements existing ecological and design-based NbS typologies. For practice, it clarifies why support strategies must be tailored to governance arrangements: state-led arrangements stabilise through mandates and long-term public budgeting; market-led delivery responds to standards, compliance regimes and investment logics; co-led forms depend on institutionalised power-sharing, intermediaries and pooled resources; and citizen- and community-led modes require enabling regulation, recognition, simplified permitting and mechanisms that stabilise stewardship beyond voluntarism.

Although archetypes were assigned retrospectively in this paper, the typology is intended for prospective use as an analytical tool. It enables early identification of misalignment by mapping veto points and decision authority, tracing whether resources can be mobilised beyond short project cycles, and testing whether stewardship is enforceable over a long-term horizon. Intergrating these metadata in NbS databases and monitoring systems would extend typologies from descriptive taxonomies to portfolio-level analytical resources that enable comparative learning, earlier corrective intervention and more plausible, equitable scaling of urban NbS as infrastructure rather than occasional pilots.

## Supplementary Information

Below is the link to the electronic supplementary material.

Supplementary file1 (PDF 254 KB)

## Data Availability

The data used in this manuscript are available in the supplementary materials.

## Footnotes

1
https://interlace-hub.com/urban-governance-atlas, accessed 10 August 2025.
