# Supplementary material for: Who leads matters: A governance-informed typology for implementing and scaling nature-based solutions
Source: Ambio. 2026 Jul 10;55(12):2996–3017. doi: 10.1007/s13280-026-02421-y (PMC13731559; doi:10.1007/s13280-026-02421-y)
Supplement: Supplementary file 1 [file 13280_2026_2421_MOESM1_ESM.pdf]

Supplementary Materials:

Who Leads Matters: A Governance-Informed Typology for Implementing and Scaling Nature-based Solutions

Kirk B. Enu [1], Christian Albert [2], Beate Jessel [1]*

[1] School of Architecture, Civil and Environmental Engineering; Laboratory of Landscape Development, Swiss Federal Technology Institute of Lausanne (EPFL); Station 16; 1015 Lausanne, Switzerland

[2] Institute of Environmental Planning, Leibniz University Hannover, Herrenhaeuser Str. 2, 30419, Hannover, Germany

Table S1. Summary of existing NbS classification frameworks, typologies and governance limitations.

Classification approach	Description	Specific typologies	Strengths	Key governance omissions	Key sources
Challenge-based	Classifies NbS by the societal or SDG-related challenges they address	Climate adaptation, biodiversity loss, air quality, disaster risk reduction, water management, social cohesion	Aligns with global development goals and funding priorities	Authority, resource control, stewardship not considered	(Anderson & Gough, 2022; Cohen-Shacham et al., 2016; Somarakis et al., 2019)
Ecosystem-based	Organised around the natural ecosystems where NbS are applied	Terrestrial, freshwater, marine, coastal, urban	Grounds NbS in ecological science; supports ecosystem service assessments	Authority, resource mobilisation, stewardship responsibilities absent	(Anderson & Gough, 2022; Cohen-Shacham et al., 2016; Sowińska-Świerkosz & Garcia, 2022)
Ecosystem change type	Based on the level and type of ecological modification or intervention	Conservation, sustainable management, restoration, ecosystem creation	Reflects ecosystem condition and restoration logic; widely used in major frameworks	Authority, resource mobilisation, stewardship not systematically addressed	(Eggermont et al., 2015; Enu & Pauleit, 2025)
Intervention modality / type	Focused on the form or technical design of NbS interventions	Green infrastructure, ecosystem-based adaptation, nature-inclusive design, permeable surfaces	Practical orientation; connects with engineering, planning, and delivery	Authority, actors, resources, and stewardship all ignored	(Anderson & Gough, 2022; Eggermont et al., 2015)
Scale-based	Considers the spatial and temporal dimensions of NbS implementation	Spatial: micro (green walls), meso (urban parks), macro (watershed restoration); Temporal: short-term (seasonal greening), long-term (forest recovery)	Aids planning across scales; supports long-term policy integration	Authority, resource control, stewardship not operationalised	(Enu & Pauleit, 2025; Kandel & Frantzeskaki, 2024; Lafortezza et al., 2018; Remme et al., 2024)
Multi-dimensional / Hybrid	Combines multiple classification logics into integrated matrices or frameworks	Typology matrices combining challenge, ecosystem, scale, and intervention type	Captures complexity; enables tailored solutions across contexts	Authority, resources, and stewardship treated as peripheral	(Somarakis et al., 2019; Sowińska-Świerkosz et al., 2024)

Table S2. Selected NbS cases for demonstrating governance-centred NbS classification. *(*On-the-ground refers to spatially implemented ecological interventions that directly modify the urban fabric and generate observable ecosystem processes. Regulatory / institutional refers to governance or policy instruments *(e.g.,* regulations, incentives or coordination programmes*)* that mandate, enable or manage NbS delivery across multiple sites rather than constituting a single spatial intervention.

No.	Case name	Location	Description	Form of implementation	Main challenge addressed	Year implemented	Sources (selected)
1	Basel Green Roof Mandate	Basel, Switzerland	Mandatory green roofs on specified building types; native species and biodiversity focus	Regulatory / institutional	Heat, biodiversity, stormwater	2002–present	https://www.urbanhabitats.org/v04n01/wildlife_pdf.pdf<br>https://climate-adapt.eea.europa.eu/en/metadata/case-studies/green-roofs-in-basel-switzerland-combining-mitigation-and-adaptation-measures-1
2	Berlin Rainwater Agency (Regenwasseragentur)	Berlin, Germany	City–NGO partnership administering stormwater permits, rebates and community outreach	Regulatory / institutional	Stormwater & green infrastructure	2012–present	https://interlace-hub.com/berlin-rainwater-agency
3	Bishan–Ang Mo Kio Park	Singapore	Transformed concrete canal into naturalized river and park under ABC (Active, Beautiful, Clean) Waters program, using bioengineering for flood resilience and public access	On-the-ground	Flooding, water quality & social benefits	2007–2012	https://www.e3s-conferences.org/articles/e3sconf/pdf/2020/54/e3sconf_icaeer2020_05060.pdf<br>https://web.mit.edu/nature/projects_14/pdfs/2014-Bishan-Ang-Mo-Kia-Park-Schaefer.pdf
4	Bosco Verticale	Milan, Italy	Residential high-rise towers with integrated vertical forests and vegetation-covered façades	On-the-ground	Heat mitigation & biodiversity	2009-2014	https://store.ctbuh.org/index.php?controller=attachment&id_attachment=32<br>https://agathon.it/agathon/article/view/294<br>https://www.mdpi.com/2075-5309/13/9/2362

5	Cape Flats Nature Project	Cape Town, South Africa	Biodiversity-centred urban greening in low-income areas, bridging social ecological infrastructure	On-the-ground	Biodiversity & climate resilience	2014–present	https://www.ecologyandsociety.org/vol17/iss2/art28/
6	Cheonggyecheon Restoration	Seoul, South Korea	Daylighting of stream formerly paved over to restore natural water flow, decrease flood risk and improve thermal comfort	On-the-ground	Flooding, heat island	2003–2005	https://www.sciencedirect.com/science/article/pii/S2210670718307716<br>https://prospernet.ias.unu.edu/wp-content/uploads/2012/09/SPC-learning-case-2_final.pdf
7	Chicago Green Alley Program	Chicago, United States	Initiated with pilot green alleys in 2001 (formal program around 2006), ongoing. Retrofitting alleys with permeable pavements, light-coloured (high albedo) surfaces, infiltration systems to reduce stormwater runoff and mitigate urban heat island effects.	On-the-ground	Stormwater & heat	2006–present	https://www.sciencedirect.com/science/article/pii/S1877705815021591?via%3Dihub#aep-article-footnote-id5<br>https://www.sciencedirect.com/science/article/pii/S0143622809000757
8	Clean River Rewards	Portland, United States	Financial offset for homeowners installing stormwater capturing features on private lots	Regulatory / institutional	Stormwater runoff	2010–present	https://www.portland.gov/bes/grants-incentives/clean-river-rewards
9	Copenhagen Klimakvarter	Copenhagen, Denmark	Neighbourhood-scale collaboration among municipal, developers and NGOs to retrofit streetscapes	On-the-ground	Heat and runoff	2018–2025	https://lup.lub.lu.se/luur/download?func=downloadFile&recordOId=8883211&fileOId=8884730
10	Guerrilla Gardening	Los Angeles, United States	Informal, citizen-led planting in neglected public spaces to cool and green streets	On-the-ground	Heat islands, greening	2000s–present	https://www.tandfonline.com/doi/full/10.1080/20004214.2024.2421577#d1e168<br>https://www.sciencedirect.com/science/article/pii/S0169204617301998?via%3Dihub

11	Hammarby Sjöstad	Stockholm, Sweden	Eco-district built through public–private partnerships with integrated stormwater ponds, green spaces and energy-efficient design.	On-the-ground	Stormwater, heat, liveability	1990s–present	https://energyinnovation.org/wp-content/uploads/2015/12/Hammarby-Sjostad.pdf<br>https://www.sciencedirect.com/science/article/pii/S0301421513002668
12	Isar Restoration	Munich, Germany	Restoration of an 8 km canalised river stretch in Munich to re-introduce floodplain dynamics, habitat connectivity, and improved flood risk management. Flood risk & ecosystem degradation. (project planning from 1995, construction 2000–2011).	On-the-ground	Flood risk & ecosystem degradation	2000–2011	https://climate-adapt.eea.europa.eu/en/metadata/case-studies/isar-plan-2013-water-management-plan-and-restoration-of-the-isar-river-munich-germany<br>https://iwaponline.com/aqua/article/71/1/42/85430/Assessment-of-NBSs-effectiveness-for-flood-risk
13	Kibera Public Spaces Project	Nairobi, Kenya	Community-designed multifunctional public spaces with flood protection and green infrastructure	On-the-ground	Flood risk & sanitation	2006–present	https://www.sciencedirect.com/science/article/pii/S2213305419300384?casa_token=r1snv4Eg0KMAAAAA:IkQhCQF55bP9gVH8TjRjuf1oC93pfOiziLlb22dVNjp5gpZVfnS8U8py1A_VZ13Zi9bF3Ayxblo
14	Masdar City Green Infrastructure & Passive Cooling	Abu Dhabi, United Arab Emirates	Eco-district masterplan integrating shaded streets, green corridors, native landscaping, and passive cooling systems to reduce urban heat and manage stormwater.	On-the-ground	Urban heat, stormwater	2006–present	https://www.witpress.com/elibrary/wit-transactions-on-ecology-and-the-environment/150/22492<br>https://www.mdpi.com/2073-445X/10/3/264
15	Medellín Green Corridors	Medellín, Colombia	Urban “green corridors” connecting 18 roads and 12 waterways with vegetation for cooling, biodiversity and public amenity.	On-the-ground	Urban heat, air pollution	2016–2019	https://www.oecd.org/content/dam/oecd/en/publications/reports/2023/10/building-systemic-climate-resilience-in-cities_a040ad72/f2f020b9-en.pdf<br>https://documents1.worldbank.org/curated/en/605601595393390081/pdf/Primer-for-Cool-Cities-Reducing-

							Excessive-Urban-Heat-with-a-Focus-on-Passive-Measures.pdf
16	Melbourne WSUD Guidelines	Melbourne, Australia	Urban water-sensitive design mandated in precincts, combining infiltration, swales, rain gardens, etc.	Regulatory / institutional	Stormwater & heat	2000s–present	https://www.sciencedirect.com/science/article/pii/S0169204610002185<br>https://www.melbournewater.com.au/sites/default/files/South-Eastern-councils-WSUD-guidelines.pdf
17	Montreal Green Alleys (Ruelles Vertes)	Montréal, Canada	Resident led transformation of alleyways into blue-green infrastructure managing runoff	On-the-ground	Stormwater, heat, inclusion	2017–present	https://www.tandfonline.com/doi/full/10.1080/02723638.2025.2536237<br>https://www.vrm.ca/en/les-ruelles-vertes-de-montreal-disparites-spatiales-et-variations-2/<br>https://www.sciencedirect.com/science/article/pii/S161886672400270X?via%3Dihub
18	Philadelphia Rain Check Program	Philadelphia, United States	Incentive scheme for homeowners installing rain barrels, gardens, permeable paving	Regulatory / institutional	Stormwater runoff	2012–present	https://www.tandfonline.com/doi/full/10.1080/13549839.2016.1191063?scroll=top&needAccess=true#d1e370
19	Prinzessinnengarten	Berlin, Germany	Community garden on vacant land hosting city-led workshops, food production, biodiversity	On-the-ground	Biodiversity & greenspace access	2009–present	https://digitalcommons.lmu.edu/cgi/viewcontent.cgi?article=1190&context=cate
20	Quito Urban Green Network	Quito, Ecuador	Municipality-led green corridor network co-developed with NGOs and resident associations to improve shade and water retention.	On-the-ground	Heat, runoff, social inclusion	2017–present	https://interlace-hub.com/urban-green-network-quito<br>https://www.ciuq.ec/Engl2/Distribucion%20accesibilidad%20y%20equidad%20territorial%20de%20las%20areas%20verdes%20urbanas%20en%20el%20Distrito%20Metropolitano%20de%20Quito.pdf

							https://academic.oup.com/jue/article/10/1/juae005/7638822
21	RiverSmart Homes	Washington, D.C., United States	District-run rebate and installation program for rain gardens, barrels, permeable surfaces, green roofs	Regulatory / institutional	Stormwater runoff	2009–present	https://www.sciencedirect.com/science/article/pii/S2666719322000206<br>https://www.eesi.org/articles/view/washington-d.c-riversmart-homes-program-helps-protect-the-chesapeake-bay-watershed
22	Rooftop Gardening	Mumbai, India	Citizen implemented rooftop food gardens to mitigate heat and improve air quality	On-the-ground	Heat, air pollution & food security	2015–present	https://digitalcommons.conncoll.edu/cgi/viewcontent.cgi?article=1012&context=envirohp
23	Room for the River	Netherlands	National programme widening floodplains and relocating dikes to restore natural river function and reduce flood risk	On-the-ground	Flooding	2006–2018	https://onlinelibrary.wiley.com/doi/10.1111/jfr3.12448<br>https://onlinelibrary.wiley.com/doi/10.1111/jfr3.12511
24	São João River Park	São Paulo, Brazil	Urban river park and floodplain restoration corridor built to manage floods, restore riparian habitat, and provide public greenspace and recreation. Focus: Flood management and social–ecological benefits.	On-the-ground	Flooding & social benefits	2009–mid 2010s	https://wwfint.awsassets.panda.org/downloads/mrwsaojoaocasestudy.pdf
25	Seattle RainWise Program	Seattle, United States	Utility-led rebate program for private rain gardens and cisterns to reduce combined sewer overflows and urban heat	Regulatory / institutional	Stormwater & heat	2011–present	https://kingcounty.gov/en/dept/dnrp/waste-services/wastewater-treatment/programs/rainwise<br>https://www.700milliongallons.org/rainwise/

26	Skyrise Greenery Incentive Scheme	Singapore	Incentives for property owners to install rooftop gardens and vertical greenery	Regulatory / institutional	Heat & biodiversity	2009–present	https://www.sciencedirect.com/science/article/pii/S1876610217301236?via%3Dihub<br>https://thescholarship.ecu.edu/server/api/core/bitstreams/72322a5b-bf1a-45b9-8f57-224523c0953a/content<br>https://www.emerald.com/sasbe/article-abstract/1/2/186/337399/Safe-design-of-skyrise-greenery-in-Singapore?redirectedFrom=fulltext
27	Songdo U Eco City	Songdo, South Korea	Private smart-city development incorporating green water bodies, urban wetlands and parks	On-the-ground	Heat, runoff, liveability	2003–present	https://www.tandfonline.com/doi/full/10.1080/15387216.2024.2309879#abstract<br>https://www.mdpi.com/2071-1050/16/5/1758
28	Suzhou Industrial Park	Suzhou, China	Eco-development zone with integrated lakes, green corridors and wetlands for stormwater and habitat	On-the-ground	Stormwater & biodiversity	1994–ongoing	https://www.sciencedirect.com/science/article/pii/S0360544213000546?via%3Dihub<br>https://www.frontiersin.org/journals/environmental-science/articles/10.3389/fenvs.2024.1361598/full<br>https://www.emerald.com/rege/article/28/4/316/364373/Suzhou-industrial-park-and-its-role-in-the-belt
29	Thames Estuary 2100 Plan	United Kingdom	A long-term adaptive flood-defence strategy for the Thames Estuary, incorporating natural-based solutions (wetlands, marshes, habitat creation) and managed realignment of estuarial systems, aimed at	Regulatory / institutional	Coastal & fluvial flooding	2001–2100	https://www.sciencedirect.com/science/article/abs/pii/S2193943821000200<br>https://www.gov.uk/government/collections/thames-estuary-2100-te2100

			managing tidal flood risk and supporting resilience.				
30	Toronto Green Roof Bylaw & Eco-Roof Incentive	Toronto, Canada	Policy requiring/encouraging green roofs on new construction and renovation to reduce runoff and improve insulation	Regulatory / institutional	Stormwater & heat	2009–present	https://qspace.library.queensu.ca/server/api/core/bitstreams/bf25e81d-6a06-4c75-afb9-ace160003244/content<br>https://www.degruyterbrill.com/document/doi/10.1515/9783110597820-006/html?lang=en&srsltid=AfmBOorG_RReEGYhRXB4gFfh-VZJXEJFjsw5lCrQfKPo4q3dE1gyJNEX
31	Urban Agriculture Program	Mexico City, Mexico	Municipal program enabling neighbourhood gardens to address local heat and water management	Regulatory / institutional	Heat, stormwater & food security	2007–2012	https://www.mdpi.com/2071-1050/12/18/7562<br>https://journals.ametsoc.org/view/journals/eint/27/1/EI-D-22-0010.1.xml
32	Vienna Cooling Streets	Vienna, Austria	District-scale cooling via planted street trees, vertical greenery and green façades	On-the-ground	Urban heat	2015–present	https://www.now-conference.org/wp-content/uploads/2020/02/304_handout_Cool-Streets_.pdf<br>https://smartcity.wien.gv.at/wp-content/uploads/sites/3/2022/05/scwr_klima_2022_web-EN.pdf
33	Vienna Courtyard Greening Program	Vienna, Austria	Housing cooperatives and city co-manage courtyard greening projects and vertical greenery for cooling and social cohesion	Regulatory / institutional	Heat & community liveability	2015–present	https://heathealth.info/wp-content/uploads/AC16590612.pdf<br>https://www.lifetreecheck.eu/getattachment/2fff480d-c43d-4bb1-ab53-3ef9b0ea6e7e/attachment%22
34	Vitoria Gasteiz Green Belt	Vitoria Gasteiz, Spain	A peri-urban greenbelt of wetlands, ecological corridors and restored natural areas around the city for flood regulation (hydrological functionality) and biodiversity enhancement.	On-the-ground	Flood prevention & biodiversity	1990s–present	https://www.mdpi.com/2073-445X/12/8/1534<br>https://pocacito.eu/sites/default/files/UrbanGreenBelt_VG.pdf

35	Wuhan Sponge City Programme	Wuhan, China	City-wide nature-based solutions network for stormwater infiltration, green space and retention/detention ponds; stormwater & urban flood mitigation.	Regulatory / institutional	Stormwater & urban flood mitigation	2015–present	https://www.tandfonline.com/doi/full/10.1080/07900627.2017.1373637 https://urbantransitions.global/wp-content/uploads/2020/03/Building-climate-resilience-and-water-security-in-cities-lessons-from-the-Sponge-City-of-Wuhan-China-final.pdf

Note: All cases listed remain active in some capacity, either through ongoing maintenance, scaling or program adaptation. Vitoria-Gasteiz Green Belt and Suzhou Industrial Park began before 2000 but were retained due to substantive governance and NbS expansions in post-2000 institutional contexts.

Table S3. NbS cases across the implementation leadership levers.

Case	Initiation	Decision Authority	Resource Control	Stewardship	Archetype
Basel Green Roof Mandate	Externally introduced	Centralised formal	Distributed	Individual/household	State-led
Berlin Rainwater Agency	Externally introduced	Hybrid authority	Distributed	Hybrid/shared	Co-led
Bishan–Ang Mo Kio Park	Externally introduced	Centralised formal	Centralised	Institutionalised public	State-led
Bosco Verticale	Externally introduced	Centralised formal	Centralised	Institutionalised private	Market-led
Cape Flats Nature Project	Externally introduced	Decentralised/shared	Pooled	Unclear/voluntary	Community-led
Cheonggyecheon	Externally introduced	Centralised formal	Centralised	Institutionalised public	State-led
Chicago Green Alley	Externally introduced	Centralised formal	Distributed	Hybrid/shared	State-led
Clean River Rewards	Externally introduced	Centralised formal	Distributed	Individual/household	Citizen-led
Copenhagen Klimakvarter	Externally introduced	Decentralised/shared	Pooled	Hybrid/shared	Co-led
Guerrilla Gardening	Locally demanded	Centralised formal	Distributed	Hybrid/shared	Citizen-led
Hammarby Sjöstad	Externally introduced	Decentralised/shared	Pooled	Hybrid/shared	Co-led
Isar Restoration	Locally demanded	Decentralised/shared	Pooled	Hybrid/shared	State-led
Kibera Public Space	Co-produced	Decentralised/shared	Pooled	Community/collective	Community-led
Masdar City	Externally introduced	Centralised formal	Centralised	Institutionalised private	Market-led
Medellín Green Corridors	Externally introduced	Centralised formal	Centralised	Hybrid/shared	Co-led
Melbourne WSUD	Externally introduced	Centralised formal	Distributed	Hybrid/shared	Market-led
Montreal Green Alleys	Locally demanded	Decentralised/shared	Pooled	Community/collective	Community-led
Philadelphia Rain Check	Externally introduced	Centralised formal	Distributed	Individual/household	Citizen-led
Prinzessinnengarten	Locally demanded	Informal/negotiated	Volunteer/informal	Community/collective	Community-led
Quito Urban Green Network	Externally introduced	Centralised formal	Pooled	Hybrid/shared	Co-led
RiverSmart Homes	Externally introduced	Centralised formal	Distributed	Individual/household	Citizen-led
Rooftop Gardening (Mumbai)	Hybrid/iterative	Informal/negotiated	Distributed	Individual/household	Citizen-led
Room for the River	Externally introduced	Decentralised/shared	Centralised	Hybrid/shared	State-led
São João River Park	Externally introduced	Centralised formal	Pooled	Unclear/voluntary	State-led
Seattle RainWise	Externally introduced	Centralised formal	Distributed	Individual/household	Co-led
Skyrise Greenery	Externally introduced	Centralised formal	Distributed	Individual/household	Citizen-led
Songdo U-Eco City	Externally introduced	Decentralised/shared	Pooled	Hybrid/shared	Market-led
Suzhou Industrial Park	Externally introduced	Decentralised/shared	Pooled	Hybrid/shared	Market-led
Thames Estuary 2100	Externally introduced	Decentralised/shared	Pooled	Hybrid/shared	State-led
Toronto Green Roof	Externally introduced	Decentralised/shared	Distributed	Individual/household	Market-led
Huertos Urbanos	Co-produced	Centralised formal	Distributed	Community/collective	Community-led
Vienna Cooling Streets	Externally introduced	Centralised formal	Centralised	Institutionalised public	State-led
Vienna Courtyard Greening	Co-produced	Centralised formal	Pooled	Community/collective	Co-led
Vitoria-Gasteiz Green Belt	Externally introduced	Centralised formal	Pooled	Hybrid/shared	State-led

Wuhan Sponge City	Externally introduced	Centralised formal	Pooled	Hybrid/shared	State-led

Table S4. Alignment of selected NbS cases with governance archetypes and traditional classification dimensions.

No.	Case name	Governance archetype	Challenge-based	Ecosystem-based	Ecosystem change type	Intervention modality / type	Spatial scale	Temporal scale
1	Basel Green Roof Mandate	State-led	Disaster risk reduction / Water management	Urban terrestrial	Sustainable management	Green infrastructure	Micro	Long-term
2	Berlin Rainwater Agency (Regenwasseragentur)	Co-led	Climate adaptation / Heat mitigation	Urban terrestrial	Sustainable management	Ecosystem-based management	Micro	Long-term
3	Bishan–Ang Mo Kio Park	State-led	Disaster risk reduction / Water management	Hybrid freshwater–urban	Sustainable management	Green infrastructure	Meso	Medium-term
4	Bosco Verticale	Market-led	Biodiversity conservation	Urban terrestrial	Sustainable management	Green infrastructure	Micro	Long-term
5	Cape Flats Nature Project	Community-led	Biodiversity conservation	Urban terrestrial	Conservation	Green infrastructure	Macro	Medium-term
6	Cheonggyecheon Stream Restoration	State-led	Disaster risk reduction / Water management	Hybrid freshwater–urban	Restoration	Restoration	Macro	Medium-term
7	Chicago Green Alley Program	State-led	Disaster risk reduction / Water management	Urban terrestrial	Sustainable management	Green infrastructure	Micro	Medium-term
8	Clean River Rewards	Citizen-led	Disaster risk reduction / Water management	Urban terrestrial	Sustainable management	Green infrastructure	Micro	Medium-term
9	Copenhagen Klimakvarter	Co-led	Disaster risk reduction / Water management	Urban terrestrial	Creation	Hybrid (Ecosystem-based adaptation / Green infrastructure)	Micro	Medium-term
10	Guerrilla Gardening (Los Angeles)	Citizen-led	Food security	Urban terrestrial	Sustainable management	Green infrastructure	Meso	Long-term
11	Hammarby Sjöstad	Co-led	Multiple challenges	Urban terrestrial	Sustainable management	Green infrastructure	Micro	Medium-term
12	Isar Restoration	State-led	Disaster risk reduction / Water management	Freshwater	Restoration	Restoration	Macro	Long-term

13	Kibera Public Space Project	Community-led	Disaster risk reduction / Water management	Urban terrestrial	Creation	Green infrastructure	Macro	Long-term
14	Masdar City Green Infrastructure & Passive Cooling	Market-led	Climate adaptation / Heat mitigation	Urban terrestrial	Sustainable management	Green infrastructure	Micro	Long-term
15	Medellín Green Corridors	Co-led	Biodiversity conservation	Urban terrestrial	Sustainable management	Green infrastructure	Meso	Long-term
16	Melbourne WSUD Guidelines	Market-led	Disaster risk reduction / Water management	Hybrid freshwater–urban	Sustainable management	Ecosystem-based management	Micro	Long-term
17	Montreal Green Alleys (Ruelles Vertes)	Community-led	Biodiversity conservation	Urban terrestrial	Sustainable management	Green infrastructure	Micro	Long-term
18	Philadelphia Rain Check Program	Community-led	Disaster risk reduction / Water management	Urban terrestrial	Sustainable management	Ecosystem-based management	Meso	Long-term
19	Prinzessinnengarten	Community-led	Biodiversity conservation	Urban terrestrial	Sustainable management	Green infrastructure	Meso	Medium-term
20	Quito Urban Green Network (Red Verde Urbana)	Co-led	Biodiversity conservation	Urban terrestrial	Sustainable management	Green infrastructure	Meso	Long-term
21	RiverSmart Homes	Citizen-led	Disaster risk reduction / Water management	Freshwater	Sustainable management	Green infrastructure	Meso	Medium-term
22	Rooftop Gardening – Fresh & Local	Citizen-led	Food security	Urban terrestrial	Sustainable management	Green infrastructure	Micro	Medium-term
23	Room for the River	State-led	Disaster risk reduction / Water management	Freshwater	Restoration	Restoration	Meso	Medium-term
24	São João River Park (Projeto Iguaçu)	State-led	Disaster risk reduction / Water management	Hybrid freshwater–urban	Restoration	Restoration	Meso	Long-term
25	Seattle RainWise Program	Co-led	Multiple challenges	Urban terrestrial	Sustainable management	Green infrastructure	Micro	Medium-term
26	Skyrise Greenery Incentive Scheme	Citizen-led	Social cohesion / Equity	Urban terrestrial	Sustainable management	Green infrastructure	Micro	Medium-term
27	Songdo U-Eco City	Market-led	Multiple challenges	Urban terrestrial	Creation	Green infrastructure	Meso	Medium-term
28	Suzhou Industrial Park	Market-led	Multiple challenges	Urban terrestrial	Sustainable management	Green infrastructure	Meso	Medium-term

29	Thames Estuary 2100 Plan (TE2100)	State-led	Disaster risk reduction / Water management	Coastal	Combined creation and sustainable management	Ecosystem-based management	Macro	Long-term
30	Toronto Green Roof Bylaw & Eco-Roof Incentive	Market-led	Disaster risk reduction / Water management	Urban terrestrial	Creation	Green infrastructure	Micro	Medium-term
31	Urban Agriculture Program (Huerto Roma Verde)	Community-led	Food security	Urban terrestrial	Sustainable management	Green infrastructure	Micro	Medium-term
32	Vienna Cooling Streets (Coole Straßen)	State-led	Climate adaptation / Heat mitigation	Urban terrestrial	Sustainable management	Green infrastructure	Micro	Medium-term
33	Vienna Courtyard Greening Program	Co-led	Multiple challenges	Urban terrestrial	Sustainable management	Green infrastructure	Micro	Long-term
34	Vitoria–Gasteiz Green Belt	State-led	Disaster risk reduction / Water management	Hybrid freshwater–urban	Sustainable management	Green infrastructure	Meso	Long-term
35	Wuhan Sponge City Program	State-led	Disaster risk reduction / Water management	Hybrid freshwater–urban	Creation	Green infrastructure	Micro	Short-term

Table S5. Descriptive statistics of cases.

Dimension	Variable	Category	n	%
Case characteristics	Form of implementation	On-the-ground	23	65.7
		Regulatory / institutional	12	34.3
	Start period	< 2000	3	8.6
		2000–2005	7	20.0
		2006–2010	13	37.1
		2011–2015	8	22.9
		≥ 2016	4	11.4
Challenges addressed (non-mutually exclusive)	Main challenge	Urban heat	20	57.1
		Stormwater / runoff	18	51.4
		(Coastal) flooding	9	25.7
		Social inclusion / liveability	8	22.9
		Biodiversity	7	20.0
		Air pollution	2	5.7
		Food security	2	5.7
		Water quality	1	2.9
		Sanitation	1	2.9
		Ecosystem restoration	1	2.9
		Climate resilience	1	2.9

Table S6. Inter-coder reliability (Cohen's κ)

Variable	Full dataset (all variables)	Constitutive (full)	Constitutive (random n=100)	Full dataset (random n=100)
Initiation	0.925	—	—	—
Decision Authority	0.767	0.767	0.669	—
Resource Control	0.781	0.781	0.730	—
Stewardship	0.686	0.686	0.657	—
Archetype	0.850	0.850	0.867	—
**Overall κ**	**0.860**	**0.832**	**0.804**	**0.837**

Sources (table S1)

Anderson, V., & Gough, W. A. (2022). A typology of nature-based solutions for sustainable development: An analysis of form, function, nomenclature, and associated applications. *Land*, *11*(7), 1072.

Cohen-Shacham, E., Walters, G., Janzen, C., & Maginnis, S. (2016). Nature-based solutions to address global societal challenges. *IUCN: Gland, Switzerland*, *97*, 2016–2036.

Eggermont, H., Balian, E., Azevedo, J. M. N., Beumer, V., Brodin, T., Claudet, J., Fady, B., Grube, M., Keune, H., & Lamarque, P. (2015). Nature-based solutions: New influence for environmental management and research in Europe. *GAIA-Ecological Perspectives for Science and Society*, *24*(4), 243–248. https://doi.org/10.14512/gaia.24.4.9

Enu, K. B., & Pauleit, S. (2025). Moving NbS from Concept to Action in Sub-Saharan Africa: Strategic Pathways for Scaling Implementation. *Built Environment*, *51*(4).

Kandel, S., & Frantzeskaki, N. (2024). Nature-based solutions and buildings: A review of the literature and an agenda for renaturing our cities one building at a time. *Nature-Based Solutions*, 100106. https://doi.org/Nature-based%2520solutions%2520and%2520buildings:%2520A%2520review%2520of%2520the%2520literature%2520and%2520an%2520agenda%2520for%2520renaturing%2520our%2520cities%2520one%2520building%2520at

Lafortezza, R., Chen, J., Van Den Bosch, C. K., & Randrup, T. B. (2018). Nature-based solutions for resilient landscapes and cities. *Environmental Research*, *165*, 431–441. https://doi.org/10.1016/j.envres.2017.11.038

Remme, R. P., Meacham, M., Pellowe, K. E., Andersson, E., Guerry, A. D., Janke, B., Liu, L., Lonsdorf, E., Li, M., & Mao, Y. (2024). Aligning nature-based solutions with ecosystem services in the urban century. *Ecosystem Services*, *66*, 101610.

Somarakis, G., Stagakis, S., & Chrysoulakis, N. (2019). *ThinkNature Nature-Based Solutions Handbook: ThinkNature project funded by the EU Horizon 2020 research and Innovation programme under grant agreement No. 730338*. European Commission. http://dx.doi.org/10.26225/jerv-w202

Sowińska-Świerkosz, B., & Garcia, J. (2022). What are Nature-based solutions (NBS)? Setting core ideas for concept clarification. *Nature-Based Solutions*, 100009. https://doi.org/10.1016/j.nbsj.2022.100009

Sowińska-Świerkosz, B., García, J., & Wendling, L. (2024). Linkages between the concept of nature-based solutions and the notion of landscape. *Ambio*, *53*(2), 227–241.
